# Public Concern About Monitoring Twitter Users and Their Conversations to Recruit for Clinical Trials: Survey Study

**DOI:** 10.2196/15455

**Published:** 2019-10-30

**Authors:** Katja Reuter, Yifan Zhu, Praveen Angyan, NamQuyen Le, Akil A Merchant, Michael Zimmer

**Affiliations:** 1 Southern California Clinical and Translational Science Institute Keck School of Medicine University of Southern California Los Angeles, CA United States; 2 Institute for Health Promotion and Disease Prevention Research, Department of Preventive Medicine Keck School of Medicine University of Southern California Los Angeles, CA United States; 3 School of Information Studies University of Wisconsin-Milwaukee Milwaukee, WI United States; 4 Cedars Sinai Medical Center Los Angeles, CA United States; 5 Department of Computer Science Marquette University Milwaukee, WI United States

**Keywords:** AIDS, cancer, clinical research, clinical trial, crowdsourcing, ethics, HIV, HPV, infoveillance, infodemiology, informed consent, Internet, research ethics, Mechanical Turk, MTurk, monitoring, obesity, privacy, public opinion, recruitment, smoking, social media, social network, surveillance, TurkPrime, Twitter

## Abstract

**Background:**

Social networks such as Twitter offer the clinical research community a novel opportunity for engaging potential study participants based on user activity data. However, the availability of public social media data has led to new ethical challenges about respecting user privacy and the appropriateness of monitoring social media for clinical trial recruitment. Researchers have voiced the need for involving users’ perspectives in the development of ethical norms and regulations.

**Objective:**

This study examined the attitudes and level of concern among Twitter users and nonusers about using Twitter for monitoring social media users and their conversations to recruit potential clinical trial participants.

**Methods:**

We used two online methods for recruiting study participants: the open survey was (1) advertised on Twitter between May 23 and June 8, 2017, and (2) deployed on TurkPrime, a crowdsourcing data acquisition platform, between May 23 and June 8, 2017. Eligible participants were adults, 18 years of age or older, who lived in the United States. People with and without Twitter accounts were included in the study.

**Results:**

While nearly half the respondents—on Twitter (94/603, 15.6%) and on TurkPrime (509/603, 84.4%)—indicated agreement that social media monitoring constitutes a form of eavesdropping that invades their privacy, over one-third disagreed and nearly 1 in 5 had no opinion. A chi-square test revealed a positive relationship between respondents’ general privacy concern and their average concern about Internet research (*P*<.005). We found associations between respondents’ Twitter literacy and their concerns about the ability for researchers to monitor their Twitter activity for clinical trial recruitment (*P*=.001) and whether they consider Twitter monitoring for clinical trial recruitment as eavesdropping (*P*<.001) and an invasion of privacy (*P*=.003). As Twitter literacy increased, so did people’s concerns about researchers monitoring Twitter activity. Our data support the previously suggested use of the *nonexceptionalist methodology* for assessing social media in research, insofar as social media-based recruitment does not need to be considered exceptional and, for most, it is considered preferable to traditional in-person interventions at physical clinics. The expressed attitudes were highly contextual, depending on factors such as the type of disease or health topic (eg, HIV/AIDS vs obesity vs smoking), the entity or person monitoring users on Twitter, and the monitored information.

**Conclusions:**

The data and findings from this study contribute to the critical dialogue with the public about the use of social media in clinical research. The findings suggest that most users do not think that monitoring Twitter for clinical trial recruitment constitutes inappropriate surveillance or a violation of privacy. However, researchers should remain mindful that some participants might find social media monitoring problematic when connected with certain conditions or health topics. Further research should isolate factors that influence the level of concern among social media users across platforms and populations and inform the development of more clear and consistent guidelines.

## Introduction

### Background

The success of clinical trials depends on the enrollment of study participants, also referred to as research participant recruitment. Recruitment involves attracting and selecting suitable study participants. It can be conducted through different communication channels (eg, newspapers, radio, television, posters, brochures, email, and social media). Without their involvement, medical and scientific progress that benefits patients would be impossible [1-5]. A recent systematic review found that 76.1% (131/172) of randomized clinical trials discontinued due to poor recruitment [6]. There is an urgent need for innovative solutions to address the issue of underenrollment in clinical trials [1]. We wanted to assess the feasibility of using Twitter user data for enhancing clinical trial recruitment. There is growing interest in using social media data for research, which is also referred to as *infoveillance* [7,8] or *digital epidemiology* [9]. This type of social media monitoring uses insights from social media users’ activity and conversations to learn more about their attitudes and behaviors. *Active recruitment* occurs when research team members approach and interact with specific individuals to enroll them in research on the basis of pre-existing knowledge of characteristics that would make them suitable candidates for particular clinical trials [10]. We hypothesized that users’ data and their conversations derived from the social network Twitter could serve as a useful tool to identify and recruit potential participants for specific clinical trials.

In the context of the Internet and social media, user privacy is commonly considered a process of boundary management where individuals regulate disclosures in their social relationships through adjustments to the transmission and sharing of personal information online. In her theory of communication privacy management, Petronio argues that individuals are regularly engaged in decisions about disclosing or concealing private information within any given context [11]. As the Internet and social media platforms become increasingly embedded into everyday life, they introduce new flows of information that challenge privacy norms and make managing boundaries more difficult. Such dynamism is central to the notion of networked privacy, which Marwick and Boyd define as the “ongoing negotiation of contexts in a networked ecosystem in which contexts regularly blur and collapse” [12]. Additionally, Nissenbaum’s theory of contextual integrity takes context as its starting point [13]. The contextual integrity framework rests on the understanding that social interactions occur in particular contexts and that norms govern people’s expectations of how personal information should flow within a given context. Rejecting the traditional dichotomy of public versus private information, as well as the notion that a user’s preferences and decisions of privacy are independent of context, contextual integrity provides a framework for evaluating the flow of personal information between different agents; it also provides a framework for explaining why certain patterns of information flow might be acceptable in one context but viewed as problematic in another. These approaches to privacy on social media platforms prompted us to consider user expectations of appropriate information flows in the context of monitoring Twitter activity for the purpose of clinical trial recruitment.

Nearly one-quarter of American adults (22%) use Twitter [14]. Twitter users can send short messages, called *tweets*, that are limited to 280 characters [15]; they can also search for any public message and further engage with tweets (ie, they can *like*, *reply to*, and *retweet* [ie, share] them). Previous research suggested that Twitter provides a “rich and promising avenue for exploring how patients conceptualize and communicate about their specific health issues” [16] and provides an avenue for raising awareness of clinical trials and boosting enrollment [17-19].

To test the feasibility of Twitter monitoring for recruiting clinical trial participants, we decided to develop a use case for a multisite cancer study on acute myeloid leukemia (AML) with patients in remission. These patients present a uniquely challenging population to recruit for clinical studies. AML, when active, typically leads to severe symptoms and hospitalization. Hospitalized patients are more accessible to screen, identify, and recruit for clinical trials. Once AML patients have completed their consolidation chemotherapy, they only visit their doctor every 3-4 months. The clinical trial we chose for this case study was designed to recruit patients in the first 3 months after they complete their consolidation chemotherapy, precisely the time when these patients have only sporadic contact with the health care system. Traditional techniques employed during routine patient contact would not be possible for this population. Furthermore, since postremission maintenance therapy is not a routine part of clinical practice for AML, we were unlikely to receive referrals from community physicians for this clinical trial. Therefore, we sought to examine the feasibility of a social media monitoring-enabled solution.

However, in their review of the study protocol, the Central Institutional Review Board (CIRB) of the National Institute of Cancer raised concerns about the potential breach of privacy using monitoring techniques on Twitter. The CIRB committee noted the following:

Those who openly share their information via social media platforms may still have an expectation of privacy and/or be unaware of the platform’s privacy policies. To contact people after utilizing the approach of “active listening” may be perceived by some potential participants as eavesdropping on their conversations about their health... This may produce distrust and potential participants may interpret this as an invasion of their privacy even though social media is understood by many to be a public sphere. Privacy risks specific to [a Twitter user’s] diagnosis may be increased by taking part in the study. The study team, by echoing the information about an individual’s diagnosis, may amplify this information, so it’s more likely to come to the attention of the public or an employer.

We used this feedback as guidance and motivation for designing the following research study to ascertain people's attitudes and level of concern about the use of social media monitoring on Twitter for targeted clinical trial recruitment.

### Study Objective and Hypotheses

Scientists have pointed out a lack of the inclusion of public views to inform future practices in social media research and social media-enabled recruitment [20,21]. Furthermore, a recent survey about the general use of tweets in research showed a lack of awareness among Twitter users that their public tweets could be used by researchers [22]. Therefore, the objective of this study was to examine the attitudes and level of concern among the public about using Twitter for the monitoring of social media users and their conversations in order to identify and recruit clinical trial participants. We focused on a variety of health topics, including cancer, obesity, human papilloma virus (HPV), HIV/AIDS, and smoking. The reason we chose to include a range of health topics, including nontransmissible and transmissible diseases, is that it reflects the spectrum of clinical trials that are being conducted in the United States and globally. We anticipated that the level of concern might vary by disease type and should be taken into consideration when choosing the recruitment method.

This study tested four primary and three additional hypotheses related to potential privacy concerns with the use of Twitter monitoring for clinical trial recruitment (see Textbox 1). Motivated by the CIRB’s comments, we developed three hypotheses to test the CIRB’s concerns regarding the potential for Twitter users to perceive social media monitoring as invasive and a violation of privacy: see Hypotheses 1-3 in Textbox 1. Drawing from Gelinas et al, we also sought to test the validity of the *nonexceptionalist methodology* [10], which suggests that recruiting clinical trial participants online should be normalized and should not be considered exceptional compared to traditional, offline recruitment strategies: see Hypothesis 4 in Textbox 1. They argue that “social media recruitment should be evaluated in substantially the same way as more traditional analogue or ‘off-line’ recruitment.” Building from these four primary hypotheses, we sought to determine whether additional factors might impact participants’ level of concern with social media monitoring for clinical trial participant recruitment. We isolated different factors within the vignettes for further analysis (ie, the type of information being monitored, the kind of disease or health topic of the clinical trial, and the nature of the entity engaged in the monitoring): see Hypotheses 5-7 in Textbox 1.

Our results are based on the views of the public and they support the formulation of evidence-based guidelines to assist researchers and Institutional Review Board (IRB) professionals using social media in clinical research recruitment. The data contribute to the critical dialogue with the public to understand the ethical issues involved in social media-enabled research and recruitment as well as the procedural solutions that are required to protect the rights and safety of research participants.

**Hypotheses we intended to test with this research study.**

Hypothesis 1: People perceive social media monitoring on Twitter for clinical trial recruitment as eavesdropping on their conversations about their health and as an invasion of their privacy.

Hypothesis 2: Twitter users’ expectations of privacy relate to their level of concern about the use of social media monitoring for clinical trial recruitment.

Hypothesis 3: General literacy and knowledge about the Twitter platform are associated with the level of concern about the use of social media monitoring on Twitter for clinical trial recruitment.

Hypothesis 4: People’s concerns over Twitter monitoring for clinical trial recruitment are similar to more traditional, offline scenarios (eg, discretely being approached in person as the patient leaves a medical facility).

Hypothesis 5: The type of information monitored to identify and recruit individuals for clinical trials is associated with the level of concern over the use of social media monitoring on Twitter for clinical trial recruitment.

Hypothesis 6: The type of disease recruited for is associated with the level of concern over the use of social media monitoring on Twitter for clinical trial recruitment.

Hypothesis 7: The type of entity performing the monitoring is associated with the level of concern over the use of social media monitoring on Twitter for clinical trial recruitment.

## Methods

### Survey Instrument

We developed an open 39-item survey (see Multimedia Appendix 1) with the overall goal of assessing participants’ attitudes and concerns regarding the use of Twitter for monitoring social media users and their conversations to identify and recruit clinical trial participants; we used a convenience sample. The following sections report on aspects of this survey study in accordance with the Checklist for Reporting Results of Internet E-Surveys (CHERRIES) [23]. Most questions were required; however, in some cases they were optional or allowed multiple answers. We incorporated two attention-check questions to assess respondents’ attentiveness to the wording of questions and eliminated from the final dataset those respondents who failed them. We tested the survey in order to evaluate the reading level and complexity of the questions, acceptability of the instrument to participants, the respondent burden, and time needed to complete the instrument. Among the testers were two community members (promotora, ie, lay Hispanic or Latino community members who receive specialized training to provide basic health education in the community without being professional health care workers) from Los Angeles, three experts from the Community Engagement Core Group team at the Southern California Clinical and Translational Science Institute at the University of Southern California, and four graduate students from the University of Wisconsin-Milwaukee. We refined the survey instrument according to their feedback, in particular the wording of the vignettes and the true and false questions.

Using the survey, we collected the following types of information: previous use and knowledge of Twitter, general concern about Internet privacy, specific concerns about privacy related to the monitoring of Twitter activity for clinical trial recruitment, and demographic data. Clinical trials were defined for respondents in accordance with the National Institutes of Health definition for nonspecialist audiences [24]:

The goal of clinical trials is to determine if a new drug, device, or procedure works and is safe, or they can look at other aspects of care, such as improving the quality of life for people with chronic illnesses. People participate in clinical trials for a variety of reasons, for example, to help others [and] to contribute to moving science forward.

Finally, we used a set of vignettes to assess the association between the level of concern and different variables, such as the disease or health topic of the clinical trial and the entity that monitors social media user activity on Twitter.

### Participants

Eligible participants were adults, 18 years of age or older, who lived in the United States. People with and without Twitter accounts were included in the study.

### Sample and Recruitment Methods

#### Overview

We used two online methods for recruiting study participants, who made up our convenience sample: the open survey was (1) advertised on Twitter between May 23 and June 8, 2017, and (2) deployed on TurkPrime, a crowdsourcing data acquisition platform, between May 23 and June 8, 2017 [25]. Accessing large numbers of participants from the Internet is referred to as crowdsourcing.

#### Twitter Recruitment

The Twitter ads appeared as promoted tweets in users’ Twitter feeds. Twitter ads provide a number of targeting options for reaching a specific target audience. Targeting features used for ads in this study included (1) *age targeting* to adults aged 18 or older, (2) *location targeting* to the United States, (3) *language targeting* to users who understand English, and (4) *keyword and hashtag targeting* for words and hashtagged words that Twitter users have tweeted or searched for on Twitter related to four main categories. The four categories to which targeted keywords or hashtags were related were (1) social media and social media surveillance, (2) research participant recruitment and clinical trial enrolment, (3) ethics and Internet privacy, and (4) clinical research and clinical trials. Each ad included a brief description (eg, “Your opinion on social media surveillance on Twitter and for a chance at a gift card. Survey and raffle entry.”), an image related to survey taking, a request for volunteers needed, a request for providing feedback, and a link to the questionnaire. Twitter ads were posted by the principal investigator's Twitter handle (ie, @dmsci). Our recruitment target was 500 participants. The daily maximum ad budget was set at US $49 with a total budget of US $980 for the entirety of the project. Respondents on Twitter had the opportunity to enter a raffle to win one of 10 US $100 gift cards upon completion of the survey. Duplicate and fraudulent responses were identified and removed as described by Teitcher et al [26]. More specifically, we used four methods to check for duplicate and fraudulent responses: (1) we checked for inconsistent and irregular answers, (2) we assessed the survey submission time stamps and batch submissions, (3) we examined email addresses that used random English words followed by three to six random letters (eg, upgradeyhujer@gmail.com), and (4) we contacted suspected respondents via email and asked them to verify the answers to three questions included in the survey to compare their responses (ie, their first name, age, and highest education level).

#### TurkPrime Recruitment

The second sample used in this study was recruited through TurkPrime [25], a panel service that allows researchers to target specific demographic groups. Prime Panels provides researchers with access to members of a number of market research panels through a Web interface similar to Amazon’s crowdsourcing platform Mechanical Turk, which has been found to be an effective method to recruit study participants online across a wide spectrum of disciplines [27-34]. However, TurkPrime offers a proportional matching sampling approach. The study was visible to eligible participants on their dashboards. They also received an email inviting them to participate in the study. We applied a *census-matched* template provided by TurkPrime that ensured that the sample proportionally matched the US adult population, aged 18 years or older, in terms of gender, age, race, ethnicity, and US region. More specifically, target benchmarks for key demographics included the following: gender—male (49.4%) and female (50.6%); age in years—18-29 (22.4%), 30-39 (16.8%), 40-49 (16.4%), 50-59 (17.8%), 60-69 (14.0%), and 70-99 (12.6%); Hispanic—not Hispanic (84.0%) and Hispanic, Latino, or Spanish (16.0%); and ethnicity—white (78.8%), black or African American (13%), American Indian or Alaska Native (1.2%), Asian (4.8%), and some other race (2.2%). These characteristics were targeted because they were underrepresented in the Twitter study convenience sample. Upon completion of the survey, study participants received compensation in the amount that they agreed to with the market research platform through which they entered the survey. Upon successful completion of the survey’s attention-check questions, participants were also given bonuses. Bonuses serve as incentives for participation and have shown a substantial effect on data quality and the creativity of workers [35]. Target recruitment was 500 participants, for a total budget of US $3500. To ensure data protection, TurkPrime ensures the following [25]:

TurkPrime... uses transport layer security encryption (also known as HTTPS) for all transmitted data. All data access is blocked except for explicitly whitelisted IP addresses, in addition to being secured with user passwords. Furthermore, [the] data, including Access Key ID and Secret Access Key, are encrypted with AES-256 encryption, the standard adopted by the National Institute of Standards and Technology.

### Data Collection

Study data were collected and managed using Research Electronic Data Capture (REDCap), an electronic data capture tool, hosted at the University of Southern California. REDCap is a secure, Web-based application designed to support data capture for research studies, providing (1) an intuitive interface for validated data entry, (2) audit trails for tracking data manipulation and export procedures, (3) automated export procedures for seamless data downloads to common statistical packages, and (4) procedures for importing data from external sources [36].

The paid ads posted on Twitter included a link to the survey hosted on REDCap. Respondents filled out a multipage survey online on either a mobile device or desktop. On TurkPrime, each respondent was provided with a unique link to a separate survey hosted on REDCap. The datasets used for analysis were generated directly from REDCap using the platform’s reporting tools. Please see the Twitter and TurkPrime recruitment sections for further details.

### Data Cleaning

A total of 603 participants completed the survey and passed the attention-check questions in this study: 94 (15.6%) on Twitter and 509 (84.4%) on TurkPrime. Among the initial 704 respondents on Twitter alone, we used Excel filters to identify and remove 70 respondents (9.9%) who did not show correct completion of the attention-check questions and 540 respondents (76.7%) who gave fraudulent responses with unique characteristics. Regarding the fraudulent responses, all of them (1) showed the same age (ie, 22 years old); (2) were submitted about 5-10 minutes apart from each other over a period of 5 days; (3) used email addresses with a consistent pattern, namely, a random English word followed by three to six random letters (eg, upgradeyhujer@gmail.com and imageiunmed@gmail.com); and (4) were confirmed to be fraudulent when respondents were asked to verify the information provided through the survey about their first name, age, and highest degree or level of school they had completed. For each filtered entry, we manually reviewed the email address to identify fraudulent emails (ie, email addresses that included a random English word followed by three to six random letter patterns). Finally, we manually sent a message to each email address and asked the users to verify the information they provided in their survey responses. Among the initial 738 responses on TurkPrime, we removed 229 responses (31.0%) that did not show correct completion of the attention-check questions.

### Data Analysis

We did not use any methods to adjust the sample, such as weighting of items or propensity scores. We analyzed the data on two levels: (1) at the respondent level to test control variables (individual factors: level 2) and (2) at the vignette level to test independent variables (contextual factors: level 1). Survey responses were first analyzed through descriptive statistical methods to assess the distribution of participants across our dependent and independent variables, such as the degree of privacy concern and demographic factors. Next, data regarding the different levels of concern for each vignette were further analyzed using pivot tables to identify any relationships between the levels of general privacy concern and the participants’ attitudes regarding the vignettes. We defined a high level of concern as responses that indicated *Very or somewhat concerned* and a low level of concern as responses that indicated *Not too or not at all concerned*. Finally, we also analyzed the responses using descriptive and inferential statistical techniques (ie, crosstabs and chi-square tests) to determine, generally, where respondents had strong concerns regarding the use of Twitter monitoring in clinical trial recruitment and where respondents had a weaker understanding of Twitter’s functions and usage policies. In particular, we looked to see if the concern regarding the use of Twitter monitoring in clinical trial recruitment was correlated with greater or less knowledge of Twitter, the type of monitoring used, or the method of outreach to the potential recruit. We report the results in aggregated form with all individually identifying information removed.

### Institutional Review Board Review and Approval

The study was reviewed and approved by the IRB at the University of Southern California (HS-17-00348).

## Results

### Description of Participants

#### Demographics

Overall, the sample of 603 participants showed the following distribution (see Multimedia Appendix 2): 324 were male (53.7%) and 261 were female (43.3%); the majority were non-Hispanic white (421/603, 64.5%), 63 (9.7%) were Hispanic, and 66 (10.1%) were African American or black; and roughly a quarter (152/603, 25.2%) were 18-29 years of age and 107 (17.7%) were older than 60 years. The mean age of all respondents was 42.66 years (SD 16.00). Additionally, 151 (25.2%) respondents reported that their lives were affected by a chronic or rare disease.

#### Twitter Usage

We further assessed Twitter usage among the 603 survey participants (see Multimedia Appendix 3). Of the 603 respondents, 301 (49.9%) had a Twitter account at the time of the study, however, 300 valid responses were received for frequency and last time-usage questions, and 174 (28.9%) never used Twitter at all. A total of 186 out of 301 respondents (61.8%) who used Twitter had public accounts (ie, every Twitter user can view their account and messages), 199 out of 300 (66.3%) used the network at least weekly, 122 out of 300 (40.7%) used the network nearly every day, and more than half (181/300, 60.3%) had sent a Twitter message within the last week.

#### Twitter Literacy and Knowledge

We attempted to assess the level of Twitter literacy and knowledge among study participants (see Multimedia Appendix 4). Overall, 1209 of the total 3015 responses (40.10%) to the Twitter literacy questions that we collected from the 603 respondents were correct and 367 answers (12.17%) were incorrect, while nearly half of the responses (1439/3015, 47.73%) indicated that participants did not know. More specifically, 429 out of 603 respondents (71.1%) correctly answered when asked about the function of hashtags, while 138 (22.9%) did not know their function. When asked about Twitter account privacy settings, the majority of respondents (355/603, 58.9%) answered correctly, but 201 (33.3%) did not know about them. On the other hand, when asked about the automatic deletion of old Twitter messages after 1 year, 159 out of 603 respondents (26.4%) answered correctly and 385 (63.8%) did not know about this. When asked about the accessibility of public Twitter messages to unregistered Twitter visitors, 80 out of 603 respondents (13.3%) answered correctly, while 177 (29.4%) selected the wrong answer and 346 (57.4%) did not know the answer. Finally, when asked about Twitter’s search capabilities that allow software programmers to search for Twitter messages by keyword and to collect profile information about the originating Twitter account, 186 out of 603 respondents (30.9%) answered correctly, while 369 (61.2%) did not know about these capabilities.

#### General Concern About Internet Privacy

We sought to learn more about general privacy concerns associated with the use of the Internet (see Multimedia Appendix 5). Of the 603 respondents, regardless of previous Twitter usage, 409 (67.8%) expressed some level of concern about their privacy while using the Internet. When asked how concerned respondents were about people they do not know obtaining personal information about them from their social media accounts and activities, 425 (70.5%) respondents expressed some level of concern. However, when asked how concerned respondents were about posts they made on social media that can be viewed by or shared with people not within their immediate network of friends or followers, fewer people (313/603, 51.9%) expressed some level of concern. As for these posts being used by companies for promotional purposes, 310 respondents (51.4%) expressed some level of concern. In contrast, 420 (69.7%) respondents expressed some level of concern about social media companies that might share or sell their information with third parties.

#### General Concern About Internet Research and Privacy

We also assessed respondents’ concerns about Internet research activities, which pertain to the use of their Twitter data for research purposes (see Multimedia Appendix 6). We found that 252 of the 603 respondents (41.8%) expressed some level of concern regarding researchers’ ability to send untargeted tweets visible to all their followers with a link for more information on how to participate in a clinical trial. Fewer respondents (226/603, 37.5%) expressed some level of concern about researchers noticing trending topics or hashtags related to health conditions, such as #Diabetes, #LungCancer, or #HeartDisease, and sending untargeted Twitter messages that include a link to more information on how to participate in a clinical trial, using the same hashtag. When asked how concerned they were about researchers actively monitoring users’ Twitter activity to identify and contact potential participants for clinical trials based on the users’ previous messages, 293 out of 603 respondents (48.6%) expressed some level of concern. However, fewer respondents (243/603, 40.3%) expressed some level of concern about researchers using paid Twitter advertisements (eg, *sponsored tweets*) to try to increase the likelihood that a clinical trial recruitment message gets seen by as many individuals as possible. Finally, 259 out of 603 respondents (43.0%) expressed some level of concern about Twitter keeping track of whether they clicked on a Twitter recruitment message related to a health study, for example, “Seeking participants for a #Cancer study.”

### Hypotheses Assessment

#### Hypothesis 1

Hypothesis 1 states that social media monitoring on Twitter for clinical trial recruitment is perceived as eavesdropping and an invasion of privacy.

To gauge respondents’ overall perception of Twitter monitoring for clinical trial recruitment, we tested the language as stated by the CIRB that *active listening* may be perceived by participants as *eavesdropping* on their conversations about their health (see Multimedia Appendix 7). When asked about monitoring of public Twitter conversations by medical researchers to identify and recruit potential clinical trial participants, 269 of 603 respondents (44.7%) considered it eavesdropping, while 333 (55.3%) did not consider it eavesdropping or did not know. Out of 603 respondents, 259 (43.0%) thought the monitoring was an invasion of their privacy, while 344 (57.0%) did not consider it an invasion of privacy or did not know. Finally, 235 of 603 respondents (39.0%) thought the monitoring was a potential breach of confidentiality, while 368 (61.1%) did not consider it a breach of confidentiality or did not know.

We isolated responses for only those respondents (409/603, 67.8%) who expressed some level of general concern about their privacy while using the Internet; we combined *Very concerned* with *Somewhat concerned* responses. These respondents’ overall opinions regarding the questions about eavesdropping, privacy, and confidentiality revealed slightly greater privacy concerns than the entire population. As reported in Multimedia Appendix 8, out of 409 respondents, 199 (48.8%) considered Twitter monitoring as eavesdropping, 202 (49.4%) considered it an invasion of their privacy, and 180 (44.0%) thought that it could jeopardize confidentiality.

We also examined the responses of those participants (178/603, 29.5%) who expressed little or no general concern about their overall privacy while using the Internet; this allowed us to assess whether those with little general privacy concern might still have elevated privacy concern about Twitter monitoring. Those with lower general Internet privacy concern indicated lower concern in response to the questions about eavesdropping, privacy, and confidentiality (see Multimedia Appendix 8). Similarly, fewer respondents with active Twitter accounts (199/603, 33.0%) indicated concerns with Twitter monitoring compared to the overall population (see Multimedia Appendix 7).

#### Hypothesis 2

Hypothesis 2 states that the expectation of Internet privacy relates to the level of concern about Internet research and Twitter monitoring for clinical trial recruitment.

We wanted to gauge whether the presence of general Internet privacy concern is related to increased concern about Internet research (see Multimedia Appendix 9). Therefore, we isolated responses for only those respondents (409/603, 67.8%) who expressed some level of general concern about their privacy while using the Internet—we combined *Very concerned* with *Somewhat concerned* responses—and compared them to the entire population reported in Multimedia Appendix 6. These respondents showed higher levels of general Internet research privacy concern. For example, 235 out of 409 respondents (57.5%) indicated concern about researchers actively monitoring Twitter to identify and contact potential participants for clinical trials, compared to only 293 respondents out of the entire population of 603 (48.6%).

Isolating for only those respondents (178/603, 29.5%) who expressed little or no general privacy concern, we found that this population generally had lower levels of Internet research privacy concern (see Multimedia Appendix 9). For example, only 55 of 178 respondents (30.9%) indicated concern about researchers actively monitoring Twitter activity to identify and contact potential clinical trial participants, compared to 293 respondents out of the entire population of 603 (48.6%). Similarly, only 32 of 178 respondents (18.0%) showed concern about researchers’ monitoring of hashtags in tweets, generally, compared to 244 respondents out of the entire population of 603 (40.5%) and 206 of the 409 respondents (50.4%) with high privacy concerns. A chi-square test was used to explore whether there is a relationship between respondents’ general privacy concerns and their average concerns about Internet research. The test, taking into account the population of 603 participants, revealed a statistically significant relationship between these variables: χ^2^_16_=143.0*, P*<.005. We then stratified responses based on Twitter use to assess whether active users of the social media platform expressed different levels of privacy concern regarding the use of Twitter for research purposes (see Multimedia Appendix 9). Respondents with active Twitter accounts (199/603, 33.0%) who indicated that they used the platform once a week or more reported lower levels of general Internet research privacy concern compared to the entire population. Our data suggest that being an active Twitter user might impact the levels of privacy concern expressed regarding Twitter-based Internet research activities.

Finally, we stratified the responses to the Twitter-monitoring vignettes (see Multimedia Appendix 10) based on respondents’ overall levels of Internet privacy concern and whether they are active Twitter users. We analyzed each vignette’s subquestions, isolating responses for those who expressed some concern and those who did not. Upon analyzing responses from the 409 participants out of 603 (67.8%) who expressed some level of general concern about their privacy while using the Internet, we discovered that a larger proportion of these respondents indicated some concern regarding each of the various Twitter-monitoring vignettes compared to the entire population (see Table 1).

We also isolated responses for those respondents (178/603, 29.5%) who expressed little or no general concern about their overall privacy while using the Internet; this allowed us to assess whether those with little general privacy concern might still have elevated privacy concern about the types of Twitter monitoring described in the vignettes. As reported in Table 1, those with lower general privacy concern indicated much lower concern over the vignette scenarios. Similarly, fewer respondents with active Twitter accounts (199/603, 33.0%) indicated concern with the Twitter-monitoring vignettes compared to the overall population, with a majority expressing concern only for the HIV/AIDS vignette. Overall, all groups expressed the most concern for the HIV/AIDS vignette and they expressed the least concern for the smoking vignette.

Finally, we performed chi-square tests to explore whether there was a relationship between general Internet privacy concern and levels of concern expressed with each vignette. The tests revealed a statistically significant relationship in all cases (*P*<.001), as reported in Table 2.

**Table 1:** Stratified analysis of vignette scenarios for respondents who indicated that they were Very concerned or Somewhat concerned about Twitter monitoring.

Vignette	Respondents (N=603), n (%)	Respondents with high general privacy concern (n=409), n (%)	Respondents with low general privacy concern (n=178), n (%)	Respondents who were active Twitter users (n=199), n (%)
Cancer vignette	300 (49.8)	244 (59.7)	51 (28.7)	75 (37.7)
Obesity vignette	299 (49.6)	241 (58.9)	52 (29.2)	76 (38.2)
HPV^a^ vignette	298 (49.4)	243 (59.4)	51 (28.7)	75 (37.7)
HIV/AIDS vignette	349 (57.9)	269 (65.8)	73 (41.0)	106 (53.3)
Smoking vignette	255 (42.3)	207 (50.6)	45 (25.3)	66 (33.2)

^a^HPV: human papilloma virus.

**Table 2:** Chi-square analysis of concern expressed by respondents for each vignette based on their general privacy concern.

Vignette	Number of valid cases, N	Pearson chi-square	*df*	*P* (asymptotic significance, 2-sided)
Cancer vignette	603	175.9	16	<.001
Obesity vignette	603	126.7	16	<.001
HPV^a^ vignette	603	124.4	16	<.001
HIV/AIDS vignette	603	79.6	16	<.001
Smoking vignette	603	102.5	16	<.001

^a^HPV: human papilloma virus.

#### Hypothesis 3

Hypothesis 3 states that general Twitter literacy is associated with the level of concern about the use of social media monitoring on Twitter for clinical trial recruitment.

There was a significant association (*P*=.001) between respondents’ Twitter literacy and their concerns about the ability of researchers to monitor their Twitter activity, generally, for the purpose of clinical trial recruitment (see Table 3). This relationship also indicates that as Twitter literacy increases, so do people’s concerns about researchers monitoring Twitter activity. Additionally, there was a significant association (*P*=.004) between respondents’ Twitter literacy and their concerns about researchers monitoring particular information types on Twitter (eg, hashtags, public tweets, and profile description) for the purpose of clinical trial recruitment. Overall, there was a significant association (*P*=.03) between respondents’ Twitter literacy and their overall concerns with researchers monitoring Twitter activity.

Related to the CIRB’s concerns, we also found a significant association between respondents’ Twitter literacy and whether they considered Twitter monitoring for clinical trial recruitment as eavesdropping (*P*<.001) and an invasion of privacy (*P*=.003). There was no significant association, however, between Twitter literacy and whether respondents felt that Twitter monitoring jeopardized confidentiality (*P*=.43).

**Table 3:** Chi-square analysis of concerns expressed by respondents based on their Twitter literacy.

Respondents’ concerns	Number of valid cases, N	Pearson chi-square	*df*	*P* (asymptotic significance, 2-sided)
Concern about the ability for researchers to monitor their Twitter activity, generally	536	22.7	6	.001
Concern about researchers monitoring particular information types on Twitter (eg, hashtags, public tweets, and profile description)	556	19.3	6	.004
Overall concern with researchers monitoring Twitter activity	513	7.2	2	.03
Consider Twitter monitoring for clinical trial recruitment as eavesdropping	602	38.1	4	<.001
Consider Twitter monitoring for clinical trial recruitment as an invasion of privacy	603	15.8	4	.003
Felt Twitter monitoring jeopardizes confidentiality	603	3.9	4	.43

#### Hypothesis 4

Hypothesis 4 states that there are differences in attitudes toward Twitter monitoring for clinical trial recruitment compared to a more traditional, offline scenario.

We also used the vignettes to assess the attitudes toward a more traditional, offline scenario (see Multimedia Appendix 10). We asked participants about their attitudes toward patients discretely being approached in person as they leave a medical facility. We found that out of all 603 respondents, regardless of previous Twitter usage and across all disease types, fewer than one-third would be more comfortable with a traditional, in-person request to join a clinical trial: cancer (176/603, 29.2%), obesity (161/603, 26.7%), HPV (169/603, 28.0%), HIV/AIDS (174/603, 28.9%), and smoking (161/603, 26.7%). For the respondents with greater overall general Internet privacy concern, there was no meaningful shift in the respondents’ comfort levels with having researchers recruit them as a research participant in person versus through Twitter monitoring.

#### Hypothesis 5

Hypothesis 5 states that the level of concern is associated with the type of information monitored for the purpose of identifying individuals to recruit for clinical trials.

We assessed the level of concern about the type of information medical researchers or research institutions might monitor and review in order to identify individuals for recruiting them into clinical trials (see Multimedia Appendix 6). When asked about monitoring of hashtags in tweets (ie, keywords used to organize and link conversations on Twitter, such as #SleepApnea, #Depression, or #HeartDisease), 244 of 603 respondents (40.5%) expressed some level of concern. When asked about reviewing the text of users’ public Twitter messages, out of 603 respondents, 265 (43.9%) expressed some level of concern, while 285 (47.3%) expressed some level of concern about reviewing the text of their profile description.

#### Hypotheses 6 and 7

Hypotheses 6 and 7 state that there is a level of concern associated with the type of disease recruited for and the type of entity performing the monitoring.

We used the set of vignettes (see Table 1) to further assess the association between the level of concern and the disease or health topic of the clinical trial and the entity that monitors social media user activity on Twitter. We found that of all 603 respondents, regardless of previous Twitter usage, most people expressed some level of concern in response to the scenario of researchers at a medical research university monitoring for an HIV/AIDS trial (349/603, 57.9%). We compared this to respondents with some level of concern in response to other disease topics and entities, such as cancer and a research team at a major research institution (300/603, 49.7%), obesity and scientists at a pharmaceutical company (299/603, 49.6%), HPV vaccination and a health officer at a state public health office (298/603, 49.4%), and smoking and a health officer at a local public health office (255/603, 42.3%). For most vignettes, the type of entity that conducted the research was selected as the most important factor contributing to the level of concern; for example, for the cancer vignette, 284 out of 603 respondents (47.1%) indicated that the entity was the most important factor, while for the obesity vignette it was 286 respondents (47.4%), for the HPV vignette it was 271 respondents (44.9%), and for the HIV/AIDS vignette it was 250 respondents (41.5%).

We further stratified responses for each vignette’s subquestions, isolating responses for those who expressed some concern—indicated *Very concerned* or *Somewhat concerned*—and those who expressed little or no concern—indicated *Not too concerned* or *Not concerned at all*—with the overall vignette scenario. As shown in Multimedia Appendix 11, *Who (or the entity who) is doing the Twitter monitoring* was the most common factor that impacted concern across all scenarios, regardless of whether the overall Internet privacy concern was low or high; the exception was with the HIV/AIDS scenario, where respondents who expressed overall concern noted that *The nature of the disease/medical condition being monitored* *for* was the main contributing factor. For the obesity and HPV scenarios, a noticeably larger portion of the respondents who expressed some concern also noted that the *Use of Twitter as a method in which the researchers contacted you* was also a contributing factor.

### Data Availability

All relevant data that support the findings of this study are available in the data repository figshare:

1. Responses from Twitter users: Monitoring Twitter for clinical trial recruitment [37].
2. Responses from TurkPrime workers: Monitoring Twitter for clinical trial recruitment [38].

## Discussion

### Principal Findings

Public social networks such as Twitter provide access to user information, including personal and sensitive data, without necessarily requiring an individual's knowledge or consent. While previous studies explored the unique ethical challenges of social media as a health research tool and research data source [10,20,39,40], there are only a few studies that offer users’ perspectives and public views on the use of social media monitoring as a clinical research recruitment tool [20,22]. For example, in a recent study, Fiesler et al found that the majority of surveyed Twitter users “felt that researchers should not be able to use tweets without consent” [22]. However, researchers have pointed out the need for views of the public on the subject to inform the development of ethical and regulatory guidelines and future practice [20,22].

The goal of this study was to contribute data that reflect public views of Twitter users and nonusers and to inform the scientific discourse about the use of Twitter user data for clinical trial recruitment. We discuss our findings in relation to our hypotheses (see Table 4) and contextual factors (eg, monitored information, study disease type, and monitoring entity) and conclude with potential implications for the practice.

**Table 4:** Summary of study findings by study hypothesis.

Hypotheses		Overall findings (nonstratified)
**Primary hypotheses: derived from CIRB^a^ feedback**		
	Hypothesis 1: Social media monitoring on Twitter for clinical trial recruitment is perceived as eavesdropping and as an invasion of privacy.	Not supported. While nearly half the respondents indicated agreement that social media monitoring constitutes a form of eavesdropping that invades their privacy, over one-third disagreed and nearly 1 in 5 had no opinion. Fewer respondents felt that social media monitoring jeopardizes confidentiality.
	Hypothesis 2: Twitter users’ expectations of privacy relate to their level of concern about the use of social media monitoring for clinical trial recruitment.	Supported. Chi-square tests revealed a positive relationship between respondents’ general privacy concerns and their average concerns about Internet research (N=603): χ^2^_16_=143.0, *P*<.005. Additionally, respondents who indicated some general privacy concern also generally expressed greater concern over social media monitoring, in general, as well as for each vignette scenario. Chi-square tests confirmed a statistically significant relationship between general privacy concern and concern for each vignette.
	Hypothesis 3: General literacy about the Twitter platform is associated with the level of concern about the use of social media monitoring on Twitter for clinical trial recruitment.	Supported. There was a statistically significant association (*P*=.001) between respondents’ Twitter literacy and their concerns about the ability for researchers to monitor their Twitter activity, generally, for the purpose of clinical trial recruitment. Overall, as Twitter literacy increased, so did people’s concerns about researchers monitoring Twitter activity. While there was an association between respondents’ Twitter literacy and whether they consider Twitter monitoring for clinical trial recruitment as eavesdropping or an invasion of privacy, there was no significant association with whether respondents felt Twitter monitoring jeopardizes confidentiality.
**Testing the validity of the *nonexceptionalist methodology***		
	Hypothesis 4: People’s concerns over Twitter monitoring for clinical trial recruitment are similar to those of more traditional, offline scenarios (eg, discretely approaching a patient in person as they leave a medical facility).	Supported. Most people were either indifferent, did not know, or were less comfortable with an in-person approach, regardless of previous Twitter usage and across all disease types. They did not find Twitter monitoring any more concerning than the more traditional means of clinical trial subject recruitment. Overall, the data presented here support the use of the *nonexceptionalist methodology* for assessing social media-based monitoring and recruitment.
**Factors that might impact the level of concern over social media monitoring for clinical trial recruitment**		
	Hypothesis 5: The type of information monitored for the purpose of identifying individuals to recruit for clinical trials is associated with the level of concern over the use of social media monitoring on Twitter for clinical trial recruitment.	Partially supported. While not a majority, nearly half the respondents did indicate general concern about researchers actively monitoring users’ Twitter activity to identify and contact potential participants for clinical trials. The greatest concern was related to reviewing the text of their profile description, with less concern expressed related to monitoring hashtags or the text of individual tweets.
	Hypothesis 6: The type of disease recruited for is associated with the level of concern over the use of social media monitoring on Twitter for clinical trial recruitment.	Supported. Nearly 6 out of 10 respondents expressed concern about monitoring for an HIV/AIDS trial compared to other disease topics that raised less concern, such as cancer, obesity, HPV^b^ vaccination, and smoking.
	Hypothesis 7: The nature of the entity performing social media monitoring on Twitter is associated with the level of concern over this monitoring for clinical trial recruitment.	Supported. The factor that most impacted the level of concern was the entity or person who conducted the Twitter monitoring and research. The exception was the HIV/AIDS scenario, where respondents who expressed overall concern noted that *The nature of the disease/medical condition being monitored for* was the main contributing factor.

^a^CIRB: Central Institutional Review Board.

^b^HPV: human papilloma virus.

### The Central Institutional Review Board’s Concerns

When we tested the concerns raised by the CIRB that *active listening* may be perceived by participants as *eavesdropping* on their conversations about their health, an invasion of their privacy, and a potential breach of confidentiality, we found that the majority of respondents did not share this view. While the CIRB’s concerns have some basis, with 4 in 10 respondents feeling Twitter monitoring is eavesdropping and an invasion of privacy, the concern was not widespread, even among those expressing higher levels of general online privacy concern. This suggests that while clinical researchers should be mindful that some Twitter users will be wary of being monitored for the purpose of clinical trial recruitment, these concerns should not prevent the recruitment strategy from being pursued. Tactics such as Privacy by Design [21], for example, through privacy notices and disclaimers, can be applied to achieve privacy in social media-based research recruitment. Our data also show a statistically significant relationship between respondents’ general privacy concern and their average concern about Internet-based research activities. Those who were generally more concerned about Internet privacy were also more concerned about different aspects of Twitter monitoring for trial recruitment, such as who was performing the monitoring and what information was being monitored. We found the opposite effect among those respondents who were generally less concerned about Internet privacy and who were active, frequent Twitter users. Our data suggest that being an active Twitter user might impact the level of privacy concern expressed regarding Twitter-based Internet research activities. This suggests that users who are more active online and aware of general privacy concerns are also more likely to be concerned about Twitter monitoring for clinical trial recruitment, due to a higher overall awareness of privacy and surveillance online. 

Furthermore, the CIRB committee noted that “those who openly share their information via social media platforms may still be unaware of the platforms’ privacy policies.” We found that there is a significant association between respondents’ Twitter literacy and their concerns about the ability for researchers to monitor their Twitter activity, generally, for the purpose of clinical trial recruitment. We further found a significant association between respondents’ Twitter literacy and their concerns about researchers monitoring particular information types on Twitter (eg, hashtags, public tweets, and profile description) for the purpose of clinical trial recruitment. We cannot state, however, that these concerns necessarily increase as Twitter literacy increases. Related to the CIRB’s concerns, we also found a significant association between respondents’ Twitter literacy and whether they consider Twitter monitoring for clinical trial recruitment as eavesdropping and an invasion of privacy; however, there was no significant association between Twitter literacy and whether respondents felt Twitter monitoring jeopardizes confidentiality. Overall, there is a significant association between respondents’ Twitter literacy and their overall concern with researchers monitoring Twitter activity, suggesting that the more that users understood about Twitter as a platform, the greater they were concerned about researchers monitoring their Twitter activity. This presents a challenge seen in many areas of online literacy, as confirmed in studies of Internet users, in general [41,42], as well as with social network users, in particular [22,43]. Thus, on the one hand, the more that people understand social media platforms, the more they are aware of possible privacy concerns. On the other hand, those who do not have high Twitter literacy might not be expressing concerns because they simply do not understand the potential threat.

### Testing the Nonexceptionalist Methodology

Gelinas et al suggested employing a *nonexceptionalist methodology* for assessing social media recruitment in research and “normalizing social media recruitment techniques while remaining sensitive to their potentially novel aspects” [10]. They argue that “social media recruitment should be evaluated in substantially the same way as more traditional analogue or ‘off-line’ recruitment.” This includes (1) the identification of “a more familiar off-line variant or equivalent of the social media technique being proposed,” (2) identification of substantive ethical considerations with a focus on the respect for the privacy and other interests of social media users and investigator transparency, and (3) clarification and evaluation of any aspects in which the online version differs from the more traditional offline equivalent. We used a series of vignettes to assess respondents’ attitudes toward a more traditional, offline scenario and asked them about their attitudes toward patients discretely being approached in person as they leave a medical facility. We found that, regardless of previous Twitter usage and across all disease types, most people were either indifferent, did not know, or were less comfortable with an in-person approach. This suggests that even while many respondents expressed concern over social media monitoring as eavesdropping or a potential violation of privacy, as noted above, they did not find it any more concerning than the more traditional means of clinical trial subject recruitment. In fact, our data show that less than one-third of the respondents preferred in-person recruitment over the Twitter-monitoring approach described in the vignettes. Even among those with a high level of general online privacy concern, only 38% preferred in-person recruitment. However, in-person recruitment is the current standard practice. Our findings support Gelinas et al, insofar as social media-based recruitment in itself does not need to be considered exceptional from the participant’s perspective, while researchers should also remain mindful that some participants will find it problematic.

### Additional Factors That Influence the Level of Concern 

Following Marwick and Boyd [12] and Nissenbaum [13], our findings support the notion that users frame privacy concerns in online platforms contextually and that when contexts collapse or blur, privacy concerns might emerge. Nearly half the respondents indicated general concern about researchers actively monitoring users’ Twitter activity to identify and contact potential participants for clinical trials. This suggests that, for many, a context collapse occurred that triggered some level of privacy concern; for example, information posted publicly for one reason, such as to share with one’s Twitter followers, was taken from that social context and used for a different purpose (ie, clinical trial recruitment).

Our findings further support the point previously made by Bender et al [21] that “within health information, there are gradients of sensitivity,” and certain health topics and disease types, such as cancer, may be considered less-sensitive personal health information. We found that the monitoring of Twitter user data that was related to HIV/AIDS raised the highest level of concern compared to monitoring related to cancer, HPV, obesity, or smoking. This may be partly due to the fact that HIV/AIDS is still associated with stigma [44]. Survey respondents commented as follows:

HIV a very serious and private disease... it is something that needs to be discussed in person.

On Twitter, users are using the specific language. These users have already disclosed their opinions or diagnosis. I feel like it's similar to outing someone on accident if a company were to just randomly ask people.

However, respondents also argued in favor of using Twitter monitoring for clinical trial recruitment:

If you talk about HIV/AIDS on Twitter or any social media, you have to know it's not private.

As long as the person or researcher making contact with the target is being very transparent about the source of the research and is happy to give information to verify their identity and intent, I wouldn't be alarmed or put off.

We identified additional factors that influenced the level of concern about monitoring Twitter user data for clinical trial recruitment. With the exception of the HIV/AIDS scenario as stated above, the factor that most impacted the level of concern was the type of entity or the person who conducted the research. Researchers who may use this approach should ensure investigator transparency; for example, investigators should refrain from fabricating online identities and clearly disclose the goal and design of the research [10]. In the case of monitoring Twitter user data for clinical trial recruitment, multiple messages could be used to introduce the project and main purpose of the outreach, as described by Reuter et al [45].

Finally, the form of contact on Twitter (ie, public replies versus private messages) played a more important role for the HIV/AIDS, obesity, and HPV scenarios, where a noticeably larger portion of the respondents expressed some concern. Respondents argued as follows:

This condition definitely need[s] to be addressed privately and not through a public reply.

Participant in response to the HIV/AIDS vignette

I think the public reply instead of a dm [direct message] could be embarrassing.

Participant in response to the obesity vignette

The nature of this can be very embarrassing and a public reply could be damaging.

Participant in response to the HPV vignette

This may be due to the stigma [44] associated with a disease such as HIV/AIDS and obesity or the level of controversy around a topic such as vaccination [46]. See Multimedia Appendix 12 for a broader sample of respondents’ comments in response to vignettes.

### Study Limitations

This study was limited to two populations: Twitter users and TurkPrime workers. The range of ages, education levels, and socioeconomic statuses of these populations could be more limited than those found in the general public. A total of 22% of US adults use Twitter, nearly equally among white, black, and Hispanic adults across all ages but with the highest usage among those 18-29 years of age [14]. TurkPrime workers (ie, turkers) are diverse across several demographic dimensions, such as age, gender, and income, but are not precisely representative of the United States as a whole [47]. Therefore, our findings may also not be generalizable to the monitoring of other social media platforms with different norms and privacy expectations, such as Reddit, Facebook, Instagram, Tumblr, or Snapchat. Although we expect to see similarities in public attitudes, future research will need to shed more light on how the results presented here might play out across different populations and different platforms.

Additionally, this was an exploratory study prompted by the feedback from a national research organization (ie, CIRB) and the sample size of this study was limited. More robust studies with a larger sample could yield additional insights. Finally, we acknowledge that while we chose seven hypotheses for this initial study, there are certainly other issues and variables that deserve further attention related to the subject in future studies.

### Conclusions

The data we presented here contribute to the critical dialogue with the public about the use of social media in clinical research. Public social networks such as Twitter offer the clinical research community a novel opportunity for identifying and engaging potential study participants based on user activity data. However, the availability of public social media data has led to new ethical challenges about respecting user privacy and the appropriateness of monitoring social media for clinical trial recruitment. The results of this study suggest that most users do not think monitoring Twitter for the purpose of clinical trial recruitment constitutes inappropriate surveillance or a violation of privacy. Our data further support the previously suggested use of the *nonexceptionalist methodology* for assessing social media in research, insofar as social media-based recruitment in itself does not need to be considered exceptional from the participant’s perspective and, for most, it is considered preferable to traditional in-person interventions at physical clinics. Notwithstanding these findings, researchers should also remain mindful that some participants might find social media monitoring problematic when connected with certain conditions. The expressed attitudes were highly contextual, depending on factors such as the type of disease or health topic and the entity or person who monitored users on Twitter. Further research should isolate factors that influence the level of concern among social media users across platforms and inform the development of more clear and consistent guidelines.

## Appendix

Multimedia Appendix 1 Open 39-item survey used in this study.

Multimedia Appendix 2 Respondents’ demographics.

Multimedia Appendix 3 Respondents’ Twitter usage.

Multimedia Appendix 4 Respondents’ Twitter literacy.

Multimedia Appendix 5 Respondents’ general Internet privacy concerns.

Multimedia Appendix 6 Respondents’ Internet research privacy concerns.

Multimedia Appendix 7 Respondents’ overall opinion of social media listening on Twitter for clinical trial recruitment.

Multimedia Appendix 8 Stratified analysis of the overall opinion of social media listening on Twitter for clinical trial recruitment for those responses that indicated agreement with the concerning issues of eavesdropping, invasion of privacy, and jeopardized confidentiality.

Multimedia Appendix 9 Stratified analysis of respondents who indicated “Very concerned” or “Somewhat concerned” about their privacy while using the Internet.

Multimedia Appendix 10 Respondents’ levels of concern about scenarios described in vignettes with a focus on study disease and the entity monitoring the social media user activity.

Multimedia Appendix 11 Responses to vignette subquestions, stratified based on level of overall Internet privacy concern with vignette scenario.

Multimedia Appendix 12 A select sample of respondents’ comments in response to the vignettes.

## Abbreviations

AML: acute myeloid leukemia
CHERRIES: Checklist for Reporting Results of Internet E-Surveys
CIRB: Central Institutional Review Board
HPV: human papilloma virus
IRB: Institutional Review Board
REDCap: Research Electronic Data Capture
