# Supplementary material for: Public Concern About Monitoring Twitter Users and Their Conversations to Recruit for Clinical Trials: Survey Study
Source: J Med Internet Res. 2019 Oct 30;21(10):e15455. doi: 10.2196/15455 (PMC6914244; doi:10.2196/15455)
Supplement: Multimedia Appendix 1 [file jmir_v21i10e15455_app1.pdf]

Survey

1. Have you ever used the social network Twitter?
    a. No, I have never used Twitter.
    b. I have visited the Twitter website before, but do not have an account.
    c. I have a registered account on Twitter.
    d. I previously had an account on Twitter, but deleted it.

2. [if response = 1c]
   I would say that I use Twitter…
    a. Almost never
    b. About once a month
    c. About once a week
    d. Nearly every day

3. [if response = 1c-d]
   When was the last time you sent a Twitter message (tweet)?
    a. I have never sent a Twitter message.
    b. Today
    c. Last week
    d. Last month
    e. More than a month ago

4. [if response = 1c-d]
   Is your Twitter account configured to be…
    a. Private (only those you approve can see your activity)?
    b. Public (anyone can follow you)?
    c. I don't know.

5. Generally, how concerned are you about your privacy while using the Internet?
    a. Very concerned
    b. Somewhat concerned
    c. Not too concerned
    d. Not concerned at all
    e. Don't know

[Use scale matrix] How concerned are you…

6. ... about people you do not know obtaining personal information about you from your social media account(s) and activities?
7. … about posts you make on social media being viewed or shared with people not within your immediate network of friends or followers?
8. … about posts you make on social media being used by companies for promotional purposes?
9. … that social media companies might share or sell your information with third parties?
    a. Very concerned

b. Somewhat concerned
c. Not too concerned
d. Not concerned at all
e. Don't know

Medical researchers may use Twitter for the purpose of identifying individuals to recruit into clinical trials, and to send recruitment messages. Below are some examples of how they could use Twitter for this purpose.

[Use scale matrix] How concerned are you about researchers...

10. …sending un-targeted tweets visible to all their followers with a link for more information on how to participate in a clinical trial?
11. … noticing trending topics or hashtags related to health conditions (such as #Diabetes, #LungCancer, or #HeartDisease), and sending un-targeted Twitter messages that include a link to more information on how to participate in a clinical trial, using the same hashtag?
12. …actively monitoring Twitter activity to identify and contact potential participants for clinical trials based on the content of their Twitter messages?
13. …using paid advertised Twitter messages ("sponsored tweets") to try to increase the likelihood that a clinical trial recruitment message gets seen by as many individuals as possible?
    a. Very concerned
    b. Somewhat concerned
    c. Not too concerned
    d. Not concerned at all
    e. Don't know

14. How concerned are you about Twitter keeping track of whether you click on a Twitter recruitment message related to a health study (for example "Seeking participants for a #Cancer study")?
    a. Very concerned
    b. Somewhat concerned
    c. Not too concerned
    d. Not concerned at all
    e. Don't know

[Use scale matrix] How concerned are you about the type of information medical researchers or research institutions might review for the purpose of identifying individuals to recruit for clinical trials?

15. Monitoring of hashtags in tweets (keywords used to organize and link conversations on Twitter, such as #SleepApnea, #Depression, or #HeartDisease)
16. Reviewing the text of your public Twitter messages
17. Reviewing the text of your profile description
    i. Very concerned
    ii. Somewhat concerned
    iii. Not too concerned
    iv. Not concerned at all
    v. Don't know

18. The purpose of this question is to assess your attentiveness to question wording. For this question, please mark the “Don’t know” response.
    a. I agree
    b. I disagree
    c. Don’t know

The next set of questions involve multiple vignettes – short hypothetical scenarios – that we would like you to consider. They involve examples of medical researchers monitoring Twitter activity in order to identify potential participants for clinical trials.

What are clinical trials? The goal of clinical trials is to determine if a new drug, device, or procedure works and is safe, or they can look at other aspects of care, such as improving the quality of life for people with chronic illnesses. People participate in clinical trials for a variety of reasons, for example to help others, to contribute to moving science forward.

19. A cancer research team at a major research university is looking for participants to take part in a clinical trial. They use a software tool that automatically searches the past 2 years of Twitter activity to locate accounts that mentioned “cancer” anywhere within the text of a Twitter message. Once a relevant message is identified, the research team sends a public “@reply” to the original message, asking if the user is interested in participating in the clinical trial.

    a. In general, how concerned would you be if your Twitter activity was monitored as described above for the purpose of recruitment into a clinical trial?
        i. Very concerned
        ii. Somewhat concerned
        iii. Not too concerned
        iv. Not concerned at all
        v. Don't know

    b. Which of the following factors most impacted your level of concern about the above scenario? (pick up to 2)
        i. The nature of the disease/medical condition being monitored for.
        ii. Whether the text of a Twitter message was monitored vs. a hashtag.
        iii. How far back in your Twitter history the researchers might look.
        iv. Who is doing the research.
        v. Whether a human being or a computer program is analyzing your Twitter messages.
        vi. The use of Twitter as a method in which the researchers contacted you.

    c. Considering the Twitter monitoring described above, would you be more or less comfortable if a cancer researcher determined your potential fit for a clinical trial based on your physical presence at a relevant medical facility, and then discreetly approached you in person as you were leaving the facility?
        i. More comfortable with in-person observation and request than with Twitter monitoring.
        ii. Neither more nor less comfortable.
        iii. Less comfortable with in-person observation and request than with Twitter monitoring
        iv. Don't know

d. Here you can leave a comment if you want to share more.

20. Scientists at a pharmaceutical company are looking for participants to take part in a clinical trial to test a drug designed to treat obesity. They use a software tool that allows them to automatically search the past 6 months of Twitter activity to locate accounts that mentioned “obesity”, “overweight” or “lose weight” within the text of a Twitter message. Once a relevant message is identified, the scientists send a public “@reply” to the original message, asking if the user is interested in participating in the clinical trial.

    a. In general, how concerned would you be if your Twitter activity was monitored as described above for the purpose of recruitment into a clinical trial?
        i. Very concerned
        ii. Somewhat concerned
        iii. Not too concerned
        iv. Not concerned at all
        v. Don't know

    b. Which of the following factors most impacted your level of concern about the above scenario? (pick up to 2)
        i. The nature of the disease/medical condition being monitored for
        ii. Whether the text of a Twitter message was monitored vs. a hashtag.
        iii. How far back in your Twitter history the researchers might look.
        iv. Who is doing the research.
        v. Whether a human being or a computer program is analyzing your Twitter messages.
        vi. The use of Twitter as a method in which the researchers contacted you.

    c. Considering the Twitter monitoring described above, would you be more or less comfortable if a scientist determined your potential fit for the trial based on your physical presence at a relevant medical facility, and then discreetly approached you in person as you were leaving the facility?
        i. More comfortable with in-person observation and request than with Twitter monitoring
        ii. Neither more nor less comfortable
        iii. Less comfortable with in-person observation and request than with Twitter monitoring
        iv. Don't know

    d. Here you can leave a comment if you want to share more.

21. A health officer at a state public health office is looking for participants to take part in a study to understand adoption of the HPV vaccination against cervical cancer. The health officer uses a software tool that allows them to search 2 years of Twitter activity to locate accounts that ever included the hashtag “#HPV” or “#HPVvaccine” or “cervical cancer prevention” in a Twitter message. Once identified, the health officer sends a public “@reply” to the original message, asking if the user is interested in participating in the clinical trial.

    a. In general, how concerned would you be if your Twitter activity was monitored as described above for the purpose of recruitment into a clinical trial?
        i. Very concerned

    ii. Somewhat concerned
    iii. Not too concerned
    iv. Not concerned at all
    v. Don't know

b. Which of the following factors most impacted your level of concern about the above scenario? (pick up to 2)
    i. The nature of the disease/medical condition being monitored for
    ii. Whether the text of a Twitter message was monitored vs. a hashtag.
    iii. How far back in your Twitter history the health officer might look.
    iv. Who is doing the research.
    v. Whether a human being or a computer program is analyzing your Twitter messages.
    vi. The use of Twitter as a method in which the health officer contacted you.

c. Considering the Twitter monitoring described above, would you be more or less comfortable if a health officer determined your potential fit for the trial based on your physical presence at a relevant medical facility, and then discreetly approached you in person as you were leaving the facility?
    i. More comfortable with in-person observation and request than with Twitter monitoring
    ii. Neither more nor less comfortable
    iii. Less comfortable with in-person observation and request than with Twitter monitoring
    iv. Don't know

d. Here you can leave a comment if you want to share more.

22. A research team at a major research university is looking for participants to take part in a clinical trial investigating treatment options for HIV/AIDS. A team member manually searches Twitter activity to locate accounts that used the hashtags “#HIV”, “#AIDS”, or “#MenWhoHaveSexWithMen” within the last 3 months. Once identified, the researchers send a public “@reply” to the original message, asking if the user is interested in participating in the clinical trial.

    a. In general, how concerned would you be if your Twitter activity was monitored as described above for the purpose of recruitment into a clinical trial?
        i. Very concerned
        ii. Somewhat concerned
        iii. Not too concerned
        iv. Not concerned at all
        v. Don't know

    b. Which of the following factors most impacted your level of concern about the above scenario? (pick up to 2)
        i. The nature of the disease/medical condition being monitored for
        ii. Whether the text of a Twitter message was monitored vs. a hashtag.
        iii. How far back in your Twitter history the researchers might look.
        iv. Who is doing the research.
        v. Whether a human being or a computer program is analyzing your Twitter messages.

vi. The use of Twitter as a method in which the researchers contacted you.

c. Considering the Twitter monitoring described above, would you be more or less comfortable if a researcher determined your potential fit for the trial based on your physical presence at a relevant medical facility, and then discreetly approached you in person as you were leaving the facility?
   i. More comfortable with in-person observation and request than with Twitter monitoring
   ii. Neither more nor less comfortable
   iii. Less comfortable with in-person observation and request than with Twitter monitoring
   iv. Don't know

d. Here you can leave a comment if you want to share more.

23. A health officer at a local public health office is looking for participants to take part in a study to understand the smoking habits of local citizens. The health officer uses a software tool that allows them to search the past 2 years of Twitter activity to locate accounts that mentioned words such as “smoking”, “cigarette”, “e-cigarette” or “vaping" within the text of a Twitter message. Once a relevant Twitter message is identified, the health officer sends a public “@reply” to the original message, asking if the user is interested in participating in the clinical trial.

   a. In general, how concerned would you be if your Twitter activity was monitored as described above for the purpose of recruitment into a clinical trial?
      i. Very concerned
      ii. Somewhat concerned
      iii. Not too concerned
      iv. Not concerned at all
      v. Don't know

   b. Which of the following factors most impacted your level of concern about the above scenario? (pick up to 2)
      i. The nature of the disease/medical condition being monitored for
      ii. Whether the text of a Twitter message was monitored vs. a hashtag.
      iii. How far back in your Twitter history the health officer might look.
      iv. Who is doing the research.
      v. Whether a human being or a computer program is analyzing your Twitter messages.
      vi. The use of Twitter as a method in which the researchers contacted you.

   c. Considering the Twitter monitoring described above, would you be more or less comfortable if a health officer determined your potential fit for the trial based on your physical presence at a relevant medical facility, and then discreetely approached you in person as you were leaving the facility?
      i. More comfortable with in-person observation and request than with Twitter monitoring
      ii. Neither more nor less comfortable
      iii. Less comfortable with in-person observation and request than with Twitter monitoring

iv. Don't know

d. Here you can leave a comment if you want to share more.

24. The purpose of this question is to assess your attentiveness to question wording. The sun rotates around the earth. For this question, please mark the “Strongly agree” response.
    a. Strongly disagree
    b. Strongly agree
    c. Don’t know

25. [if response = 1.c]
    Twitter offers a Privacy Policy to users that discusses how it collects, stores and shares user information. The Privacy Policy states that Twitter is primarily designed to help you share information with the world. View the full policy here: https://twitter.com/privacy?lang=en. Select the option below that best describes how closely you have read the Twitter Privacy Policy.
    a. I have never read the Privacy Policy.
    b. I have skimmed over the Privacy Policy.
    c. I have read the Privacy Policy in some detail, but not fully.
    d. I have fully read the Privacy Policy in detail.

<u>For the following questions, please select what you believe to be the correct answer.</u>

26. Including a hashtag (the ‘#’ symbol) in front of a keyword, such as #BladderCancer or #Pregnancy, is used as a way to link Twitter messages to a specific topic and make it easy for others who are interested in the same topic to find them.
    a. Yes, this is correct. [X]
    b. No, this is incorrect.
    c. I don’t know the answer to this question.

27. You can protect your Twitter messages by changing your account settings to private. Then your messages won’t be accessible to everyone.
    a. Yes, this is correct. [X]
    b. No, this is incorrect.
    c. I don’t know the answer to this question.

28. Old Twitter messages are automatically deleted from Twitter’s servers after 1 year.
    a. Yes, this is correct.
    b. No, this is incorrect. [X]
    c. I don’t know the answer to this question.

29. Unregistered visitors to Twitter can still view publicly created Twitter messages but cannot use the "search" feature of the website to view older tweets.
    a. Yes, this is correct.
    b. No, this is incorrect. [X]
    c. I don’t know the answer to this question.

30. Twitter offers a search interface that allows software programmers to search for Twitter messages by keyword and to collect profile information about the originating Twitter account.
    a. Yes, this is correct. [X]
    b. No, this is incorrect.
    c. I don’t know the answer to this question.

31. Do you consider monitoring of public Twitter conversations by medical researchers to identify potential study participants for clinical trials as <u>eavesdropping</u> on your conversations about your health?
    a. Yes, I do.
    b. No, I don’t.
    c. I don’t know.

32. Do you think medical researchers that listen to public Twitter conversations to identify potential study participants for clinical trials are <u>invading your privacy</u>?
    a. Yes, I do.
    b. No, I don’t.
    c. I don’t know.

33. Do you think medical researchers that listen to public Twitter conversations to identify potential study participants for clinical trials jeopardize confidentiality (the obligation to safeguard entrusted information from unauthorized access, use, disclosure, modification, loss, or theft)?
    a. Yes, I do.
    b. No, I don’t.
    c. I don’t know.

<u>Please tell us more about yourself.</u>

34. Is your life affected by a chronic or rare disease?
    a. Yes
    b. No
    c. I don’t want to share this information.

35. What’s your age? *(Drop-down)*

36. Are you…?
    a. Female
    b. Male
    c. Other
    d. I don’t want to share this information.

37. How do you describe yourself? (check all that apply)
    a. African American / Black
    b. American Indian / Alaska Native
    c. Asian / Pacific Islander
    d. Hispanic
    e. Middle Eastern
    f. White
    g. Other

h. I don’t want to share this information.

38. What is the highest degree or level of school you have completed?
- No schooling completed
- Nursery school to 8th grade
- Some high school, no diploma
- High school graduate, diploma or the equivalent (for example: GED)
- Some college credit, no degree
- Trade/technical/vocational training
- Associate degree
- Bachelor’s degree
- Master’s degree
- Professional degree
- Doctorate degree

Note: Question 39 appears only for respondents on Twitter (not for respondents on TurkPrime).

39. Thank you for your time! If you want to enter the raffle for one of ten $100 gift cards, please share the following information. *This information will be stored separately in a secure database, solely for the purpose of the raffle.*

- First name:
- Email:
