# Supplementary material for: Public Concern About Monitoring Twitter Users and Their Conversations to Recruit for Clinical Trials: Survey Study
Source: J Med Internet Res. 2019 Oct 30;21(10):e15455. doi: 10.2196/15455 (PMC6914244; doi:10.2196/15455)
Supplement: Multimedia Appendix 2 [file jmir_v21i10e15455_app2.pdf]

Multimedia Appendix 2: Respondents' demographics.

CHARACTERISTIC		N (%)
**Life affected by a chronic or rare disease**		
	Yes	151 (25.2%)
	No	413 (68.8%)
	I don't want to share this information	36 (6.0%)
**Age**		
	18-29	152 (25.2%)
	30-39	136 (22.6%)
	40-49	119 (19.7%)
	50-59	89 (14.8%)
	60-69	61 (10.1%)
	70-99	46 (7.6%)
**Gender**		
	Male	324 (53.7%)
	Female	261 (43.3%)
	Other	7 (1.2%)
	I don't want to share this info	11 (1.8%)
**Ethnicity/racial background** (multiple options were selectable)		
	African American / Black	66 (10.1%)
	American Indian / Alaska Native	24 (3.7%)
	Asian / Pacific Islander	42 (6.4%)
	Hispanic	63 (9.7%)
	Middle Eastern	10 (1.5%)

	White	421 (64.5%)
	Other	23 (3.5%)
	I don't want to share this information	4 (0.6%)
**Degree/level of school**		
	No schooling completed	1 (0.2%)
	Nursery school to 8th grade	5 (0.8%)
	Some high school, no diploma	17 (2.8%)
	High school graduate or equivalent	118 (19.6%)
	Some college credit, no degree	128 (21.2%)
	Trade/technical/vocational training	42 (7.0%)
	Associate degree	64 (10.6%)
	Bachelor’s degree	153 (25.4%)
	Master’s degree	56 (9.3%)
	Professional degree	8 (1.3%)
	Doctorate degree	11 (1.8%)
