# Supplementary material for: Public Concern About Monitoring Twitter Users and Their Conversations to Recruit for Clinical Trials: Survey Study
Source: J Med Internet Res. 2019 Oct 30;21(10):e15455. doi: 10.2196/15455 (PMC6914244; doi:10.2196/15455)
Supplement: Multimedia Appendix 3 [file jmir_v21i10e15455_app3.pdf]

Multimedia Appendix 3: Respondents' Twitter usage.

TWITTER USAGE		N (%)
**Previous use**		
	No, never used	174 (28.9%)
	Visited website, no account	82 (13.6%)
	Have a registered account	301 (49.9%)
	Previously, but deleted account	46 (7.6%)
**Frequency of use**		
	Almost never	48 (16.0%)
	About one per month	53 (17.7%)
	About once a week	77 (25.7%)
	Nearly every day	122 (40.7%)
	No response	1
**Last Twitter message sent**		
	I have never sent a Tweet	30 (10.0%)
	Today	81 (27.0%)
	Last week	100 (33.3%)
	Last month	30 (10.0%)
	More than a month ago	59 (19.7%)
	No response	1
**Twitter account privacy setting**		
	Private	66 (21.9%)
	Public	186 (61.8%)
	Don't know	49 (16.3%)
