# Supplementary material for: Public Concern About Monitoring Twitter Users and Their Conversations to Recruit for Clinical Trials: Survey Study
Source: J Med Internet Res. 2019 Oct 30;21(10):e15455. doi: 10.2196/15455 (PMC6914244; doi:10.2196/15455)
Supplement: Multimedia Appendix 4 [file jmir_v21i10e15455_app4.pdf]

Multimedia Appendix 4: Respondents' Twitter literacy.

TWITTER LITERACY		N (%)
**Including a hashtag (the '#' symbol) in front of a keyword, such as #BladderCancer or #Pregnancy, is used as a way to link Twitter messages to a specific topic and make it easy for others who are interested in the same topic to find them.**		
	Yes, this is correct (CORRECT)	429 (71.1%)
	No, this is incorrect	36 (6.0%)
	Don't know	138 (22.9%)
**You can protect your Twitter messages by changing your account settings to private. Then your messages won't be accessible to everyone.**		
	Yes, this is correct (CORRECT)	355 (58.9%)
	No, this is incorrect	47 (7.8%)
	Don't know	201 (33.3%)
**Old Twitter messages are automatically deleted from Twitter's servers after 1 year.**		
	Yes, this is correct	59 (9.8%)
	No, this is incorrect (CORRECT)	159 (26.4%)
	Don't know	385 (63.9%)
**Unregistered visitors to Twitter can still view publicly created Twitter messages but cannot use the "search" feature of the website to view older tweets.**		
	Yes, this is correct	177 (29.4%)
	No, this is incorrect (CORRECT)	80 (13.3%)
	Don't know	346 (57.4%)
**Twitter offers a search interface that allows software programmers to search for Twitter messages by keyword and to collect profile information about the originating Twitter account.**		
	Yes, this is correct (CORRECT)	186 (30.9%)
	No, this is incorrect	48 (8.0%)
	Don't know	369 (61.2%)
