# Supplementary material for: Public Concern About Monitoring Twitter Users and Their Conversations to Recruit for Clinical Trials: Survey Study
Source: J Med Internet Res. 2019 Oct 30;21(10):e15455. doi: 10.2196/15455 (PMC6914244; doi:10.2196/15455)
Supplement: Multimedia Appendix 5 [file jmir_v21i10e15455_app5.pdf]

Multimedia Appendix 5: Respondents' general Internet privacy concerns.

GENERAL INTERNET PRIVACY CONCERNS		N (%)
**Concern about privacy while using the Internet**		
	Very concerned	156 (25.9%)
	Somewhat concerned	253 (42.0%)
	Not too concerned	131 (21.7%)
	Not concerned at all	47 (8.0%)
	Don't know	16 (2.7%)
**Concern about people obtaining personal information about Twitter users from their social media account(s) and activities**		
	Very concerned	213 (35.3%)
	Somewhat concerned	212 (35.2%)
	Not too concerned	119 (19.7%)
	Not concerned at all	49 (8.1%)
	Don't know	10 (1.7%)
**Concern about posts users make on social media being viewed or shared with people not within their immediate network of friends or followers**		
	Very concerned	141 (23.4%)
	Somewhat concerned	172 (28.5%)
	Not too concerned	170 (28.2%)
	Not concerned at all	111 (18.4%)
	Don't know	9 (1.5%)
**Concern about posts users make on social media being used by companies for promotional purposes**		
	Very concerned	148 (24.5%)
	Somewhat concerned	162 (26.9%)
	Not too concerned	187 (31.0%)
	Not concerned at all	93 (15.4%)
	Don't know	13 (2.1%)

**Concern about social media companies sharing or selling Twitter users' information with third parties**		
	Very concerned	215 (35.7%)
	Somewhat concerned	205 (34.0%)
	Not too concerned	110 (18.2%)
	Not concerned at all	64 (10.6%)
	Don't know	9 (1.5%)
