# Supplementary material for: Public Concern About Monitoring Twitter Users and Their Conversations to Recruit for Clinical Trials: Survey Study
Source: J Med Internet Res. 2019 Oct 30;21(10):e15455. doi: 10.2196/15455 (PMC6914244; doi:10.2196/15455)
Supplement: Multimedia Appendix 6 [file jmir_v21i10e15455_app6.pdf]

Multimedia Appendix 6: Respondents' Internet research privacy concerns.

GENERAL INTERNET RESEARCH PRIVACY CONCERNS		N (%)
**Concern about researchers sending un-targeted tweets visible to all their followers with a link for more information on how to participate in a clinical trial**		
	Very concerned	106 (17.6%)
	Somewhat concerned	146 (24.2%)
	Not too concerned	178 (29.5%)
	Not concerned at all	141 (23.4%)
	Don't know	32 (5.3%)
**Concern about researchers noticing trending topics or hashtags related to health conditions (such as #Diabetes, #LungCancer, or #HeartDisease), and sending un-targeted Twitter messages that include a link to more information on how to participate in a clinical trial, using the same hashtag.**		
	Very concerned	86 (14.3%)
	Somewhat concerned	140 (23.2%)
	Not too concerned	184 (30.5%)
	Not concerned at all	165 (27.4%)
	Don't know	28 (4.6%)
**Concern about researchers actively monitoring Twitter activity to identify and contact potential participants for clinical trials**		
	Very concerned	122 (20.2%)
	Somewhat concerned	171 (28.4%)
	Not too concerned	164 (27.2%)
	Not concerned at all	110 (18.2%)
	Don't know	36 (6.0%)
**Concern about researchers using paid advertised Twitter messages ("sponsored tweets") to try to increase the likelihood that a clinical trial recruitment message gets seen by as many individuals as possible**		
	Very concerned	93 (15.4%)
	Somewhat concerned	150 (24.9%)
	Not too concerned	179 (29.7%)

	Not concerned at all	151 (25.0%)
	Don't know	30 (5.0%)
**Concern about Twitter keeping track of whether you click on a Twitter recruitment message related to a health study (for example “Seeking participants for a #Cancer study”)**		
	Very concerned	88 (14.6%)
	Somewhat concerned	171 (28.4%)
	Not too concerned	200 (33.2%)
	Not concerned at all	108 (17.9%)
	Don't know	35 (5.8%)
**Concern about monitoring of hashtags in tweets (keywords used to organize and link conversations on Twitter, such as #SleepApnea, #Depression, or #HeartDisease)**		
	Very concerned	83 (13.8%)
	Somewhat concerned	161 (26.7%)
	Not too concerned	199 (33.0%)
	Not concerned at all	134 (22.2%)
	Don't know	26 (4.3%)
**Concern about reviewing the text of users’ public Twitter messages**		
	Very concerned	108 (17.9%)
	Somewhat concerned	157 (26.0%)
	Not too concerned	186 (30.9%)
	Not concerned at all	122 (20.2%)
	Don't know	30 (5.0%)
**Concern about reviewing the text of users’ profile description**		
	Very concerned	132 (21.9%)
	Somewhat concerned	153 (25.4%)
	Not too concerned	186 (30.9%)
	Not concerned at all	108 (17.9%)
	Don't know	24 (4.0%)
