# Supplementary material for: Public Concern About Monitoring Twitter Users and Their Conversations to Recruit for Clinical Trials: Survey Study
Source: J Med Internet Res. 2019 Oct 30;21(10):e15455. doi: 10.2196/15455 (PMC6914244; doi:10.2196/15455)
Supplement: Multimedia Appendix 7 [file jmir_v21i10e15455_app7.pdf]

Multimedia Appendix 7: Respondents' overall opinion of social media listening on Twitter for clinical trial recruitment.

OVERALL OPINION ON TWITTER MONITORING FOR CLINICAL TRIAL RECRUITMENT		N (%)
**Do you consider monitoring of public Twitter conversations by medical researchers to identify potential study participants for clinical trials as eavesdropping on your conversations about your health?**		
	Yes, I do	269 (44.7%)
	No, I don't	223 (37.0%)
	Don't know	110 (18.3%)
**Do you think medical researchers that listen to public Twitter conversations to identify potential study participants for clinical trials are invading your privacy?**		
	Yes, I do	259 (43.0%)
	No, I don't	231 (38.3%)
	Don't know	113 (18.7%)
**Do you think medical researchers that listen to public Twitter conversations to identify potential study participants for clinical trials si (the obligation to safeguard entrusted information from unauthorized access, use, disclosure, modification, loss, or theft)?**		
	Yes, I do	235 (39.0%)
	No, I don't	203 (33.7%)
	Don't know	165 (27.4%)
