# Supplementary material for: Public Concern About Monitoring Twitter Users and Their Conversations to Recruit for Clinical Trials: Survey Study
Source: J Med Internet Res. 2019 Oct 30;21(10):e15455. doi: 10.2196/15455 (PMC6914244; doi:10.2196/15455)
Supplement: Multimedia Appendix 8 [file jmir_v21i10e15455_app8.pdf]

Multimedia Appendix 8: Stratified analysis of overall opinion of social media listening on Twitter for clinical trial recruitment for those responses that indicated agreement with concerning issues such as eavesdropping, invasion of privacy, and jeopardized confidentiality.

OVERALL OPINION ON TWITTER MONITORING FOR CLINICAL TRIAL RECRUITMENT	All (603)	High General Privacy Concern (409)	Low General Privacy Concern (178)	Active Twitter User (199)
**Do you consider monitoring of public Twitter conversations by medical researchers to identify potential study participants for clinical trials as eavesdropping on your conversations about your health?**	269 (41.8%)	199 (48.8%)	65 (36.5%)	74 (37.2%)
**Do you think medical researchers that listen to public Twitter conversations to identify potential study participants for clinical trials are invading your privacy?**	226 (37.5%)	202 (49.4%)	53 (29.8%)	61 (30.7%)
**Do you think medical researchers that listen to public Twitter conversations to identify potential study participants for clinical trials jeopardize confidentiality (the obligation to safeguard entrusted information from unauthorized access, use, disclosure, modification, loss, or theft)?**	293 (48.6%)	180 (44.0%)	53 (29.8%)	81 (40.7%)
