# Supplementary material for: Public Concern About Monitoring Twitter Users and Their Conversations to Recruit for Clinical Trials: Survey Study
Source: J Med Internet Res. 2019 Oct 30;21(10):e15455. doi: 10.2196/15455 (PMC6914244; doi:10.2196/15455)
Supplement: Multimedia Appendix 9 [file jmir_v21i10e15455_app9.pdf]

Multimedia Appendix 9: Stratified analysis of respondents who indicated “Very concerned” or “Somewhat concerned” about their privacy while using the Internet.

GENERAL INTERNET RESEARCH PRIVACY CONCERNS	All (603)	High General Privacy Concern (409)	Low General Privacy Concern (178)	Active Twitter User (199)
**Concern about researchers sending un-targeted tweets visible to all their followers with a link for more information on how to participate in a clinical trial**	252 (41.8%)	204 (49.9%)	43 (24.2%)	74 (37.2%)
**Concern about researchers noticing trending topics or hashtags related to health conditions (such as #Diabetes, #LungCancer, or #HeartDisease), and sending un-targeted Twitter messages that include a link to more information on how to participate in a clinical trial, using the same hashtag.**	226 (37.5%)	186 (45.5%)	32 (18.0%)	61 (30.7%)
**Concern about researchers actively monitoring Twitter activity to identify and contact potential participants for clinical trials**	293 (48.6%)	235 (57.5%)	55 (30.9%)	81 (40.7%)
**Concern about researchers using paid advertised Twitter messages (“sponsored tweets”) to try to increase the likelihood that a clinical trial recruitment message gets seen by as many individuals as possible**	243 (40.3%)	201 (49.1%)	38 (21.3%)	69 (34.7%)
**Concern about Twitter keeping track of whether you click on a Twitter recruitment message related to a health study (for example “Seeking participants for a #Cancer study”)**	259 (43.0%)	214 (52.5%)	44 (24.7%)	66 (33.2%)
**Concern about monitoring of hashtags in tweets (keywords used to organize and link conversations on Twitter, such as #SleepApnea, #Depression, or #HeartDisease)**	244 (40.0%)	206 (50.4%)	32 (18.0%)	66 (33.2%)
**Concern about reviewing the text of users’ public Twitter messages**	265 (43.9%)	218 (53.5%)	42 (23.6%)	73 (36.7%)
**Concern about reviewing the text of users’ profile description**	285 (47.3%)	225 (55.0%)	53 (29.8%)	79 (39.7%)
