# Supplementary material for: Public Concern About Monitoring Twitter Users and Their Conversations to Recruit for Clinical Trials: Survey Study
Source: J Med Internet Res. 2019 Oct 30;21(10):e15455. doi: 10.2196/15455 (PMC6914244; doi:10.2196/15455)
Supplement: Multimedia Appendix 10 [file jmir_v21i10e15455_app10.pdf]

Multimedia Appendix 10: Respondents' concern about scenarios described in vignettes with a focus on study disease and entity that monitors social media user activity.

CONCERN EXPRESSED IN RESPONSE TO VIGNETTES		N (%)
**Cancer** vignette: A *cancer research team* at a major *research university* is looking for participants to take part in a clinical trial. They use a software tool that automatically search the past 2 years of Twitter activity to locate accounts that mentioned "*cancer*" anywhere within the text of a Twitter message. Once a relevant message is identified, the research team sends a public "@reply" to the original message, asking if the user is interested in participating in the clinical trial.		
**Concern about users' Twitter activity being monitored as described above for the purpose of recruitment into a clinical trial**		
	Very concerned	110 (18.2%)
	Somewhat concerned	190 (31.5%)
	Not too concerned	181 (30.0%)
	Not concerned at all	102 (16.9%)
	Don't know	20 (3.3%)
**Factors that most impacted level of concern about the above scenario** (respondents could select up to 2)		
	Nature of the disease/medical condition being monitored for	163 (27.0%)
	Whether the text of a Twitter message was monitored vs. a hashtag	152 (25.2%)
	How far back in your Twitter history the researchers might look	167 (27.7%)
	Who is doing the research	284 (47.1%)
	Whether a human being or a computer program is analyzing your Twitter messages	148 (24.5%)
	Use of Twitter as a method in which the	162 (26.9%)

	researchers contacted you	
**Comfort level if a researcher approached them in person at a relevant medical facility**		
	More comfortable in-person	176 (29.2%)
	Neither more or less comfort	209 (34.7%)
	Less comfortable in-person	137 (22.8%)
	Don't know	81 (13.4%)
**Obesity** vignette: *Scientists at a pharmaceutical company* are looking for participants to take part in a clinical trial to test a drug designed to treat *obesity*. They use a software tool that allows them to automatically search the past 6 months of Twitter activity to locate accounts that mentioned “obesity”, “overweight” or “lose weight” within the text of a Twitter message. Once a relevant message is identified, the scientists send a public “@reply” to the original message, asking if the user is interested in participating in the clinical trial.		
**Concern about users’ Twitter activity being monitored as described above for the purpose of recruitment into a clinical trial**		
	Very concerned	112 (18.6%)
	Somewhat concerned	187 (31.0%)
	Not too concerned	181 (30.0%)
	Not concerned at all	99 (16.4%)
	Don't know	24 (4.0%)
**Factors that most impacted level of concern about the above scenario** (respondents could select up to 2)		
	Nature of the disease/medical condition being monitored for	169 (28.0%)
	Whether the text of a Twitter message was monitored vs. a hashtag	149 (24.7%)
	How far back in your Twitter history the researchers might look	147 (24.4%)
	Who is doing the research	286 (47.4%)

	Whether a human being or a computer program is analyzing your Twitter messages	123 (20.4%)
	Use of Twitter as a method in which the researchers contacted you	187 (31.0%)
**Comfort level if a researcher approached them in person at a relevant medical facility**		
	More comfortable in-person	161 (26.7%)
	Neither more or less comfort	225 (37.3%)
	Less comfortable in-person	144 (23.9%)
	Don't know	73 (12.1%)
**HPV** vignette: A health officer at a state public health office is looking for participants to take part in a study to understand adoption of the HPV vaccination against cervical cancer. The health officer uses a software tool that allows them to search 2 years of Twitter activity to locate accounts that ever included the hashtag "#HPV" or "#HPVvaccine" or "cervical cancer prevention" in a Twitter message. Once identified, the health officer sends a public "@reply" to the original message, asking if the user is interested in participating in the clinical trial.		
**Concern about users' Twitter activity being monitored as described above for the purpose of recruitment into a clinical trial**		
	Very concerned	123 (20.4%)
	Somewhat concerned	175 (29.0%)
	Not too concerned	175 (29.0%)
	Not concerned at all	101 (16.8%)
	Don't know	29 (4.8%)
**Factors that most impacted level of concern about the above scenario** (respondents could select up to 2)		
	Nature of the disease/medical condition being monitored for	202 (33.5%)
	Whether the text of a Twitter message was monitored vs. a hashtag	162 (26.9%)
	How far back in your Twitter history the	126 (20.9%)

	researchers might look	
	Who is doing the research	271 (44.9%)
	Whether a human being or a computer program is analyzing your Twitter messages	122 (20.2%)
	Use of Twitter as a method in which the researchers contacted you	173 (28.7%)
**Comfort level if a researcher approached them in person at a relevant medical facility**		
	More comfortable in-person	169 (28.0%)
	Neither more or less comfort	233 (38.6%)
	Less comfortable in-person	136 (22.6%)
	Don't know	65 (10.8%)
**HIV/AIDS** vignette: A research team at a major research university is looking for participants to take part in a clinical trial investigating treatment options for *HIV/AIDS*. A team member manually searches Twitter activity to locate accounts that used the hashtags "#HIV", "#AIDS", or "#SexwithMen" within the last 3 months. Once identified, the researchers send a public "@reply" to the original message, asking if the user is interested in participating in the clinical trial.		
**Concern about users' Twitter activity being monitored as described above for the purpose of recruitment into a clinical trial**		
	Very concerned	174 (28.9%)
	Somewhat concerned	175 (29.0%)
	Not too concerned	132 (21.9%)
	Not concerned at all	96 (15.9%)
	Don't know	26 (4.3%)
**Factors that most impacted level of concern about the above scenario** (respondents could select up to 2)		
	Nature of the disease/medical condition being monitored for	243 (40.3%)
	Whether the text of a Twitter message was	154 (25.5%)

	monitored vs. a hashtag	
	How far back in your Twitter history the researchers might look	133 (22.1%)
	Who is doing the research	250 (41.5%)
	Whether a human being or a computer program is analyzing your Twitter messages	127 (21.1%)
	Use of Twitter as a method in which the researchers contacted you	190 (31.5%)
**Comfort level if a researcher approached them in person at a relevant medical facility**		
	More comfortable in-person	174 (28.9%)
	Neither more or less comfort	213 (35.3%)
	Less comfortable in-person	156 (25.9%)
	Don't know	60 (10.0%)
**Smoking** vignette: A health officer at a local public health office is looking for participants to take part in a study to understand the smoking habits of local citizens. The health officer uses a software tool that allows them to search the past 2 years of Twitter activity to locate accounts that mentioned words such as “smoking”, “cigarette”, “e-cigarette” or “vaping" within the text of a Twitter message. Once a relevant Twitter message is identified, the health officer sends a public “@reply” to the original message, asking if the user is interested in participating in the clinical trial.		
**Concern about users’ Twitter activity being monitored as described above for the purpose of recruitment into a clinical trial**		
	Very concerned	91 (15.1%)
	Somewhat concerned	164 (27.2%)
	Not too concerned	190 (31.5%)
	Not concerned at all	142 (23.6%)
	Don't know	16 (2.7%)
**Factors that most impacted level of concern about the above scenario** (respondents could select up to 2)		

	Nature of the disease/medical condition being monitored for	163 (27.0%)
	Whether the text of a Twitter message was monitored vs. a hashtag	146 (24.2%)
	How far back in your Twitter history the researchers might look	143 (24.7%)
	Who is doing the research	269 (44.6%)
	Whether a human being or a computer program is analyzing your Twitter messages	123 (20.4%)
	Use of Twitter as a method in which the researchers contacted you	173 (28.7%)
**Comfort level if a researcher approached them in person at a relevant medical facility**		
	More comfortable in-person	161 (26.7%)
	Neither more or less comfort	267 (44.3%)
	Less comfortable in-person	122 (20.3%)
	Don't know	52 (8.4%)
