# Supplementary material for: Public Concern About Monitoring Twitter Users and Their Conversations to Recruit for Clinical Trials: Survey Study
Source: J Med Internet Res. 2019 Oct 30;21(10):e15455. doi: 10.2196/15455 (PMC6914244; doi:10.2196/15455)
Supplement: Multimedia Appendix 11 [file jmir_v21i10e15455_app11.pdf]

Multimedia Appendix 11: Responses to vignette sub-questions, stratified based on level of overall Internet privacy concern with vignette scenario.

CONCERN EXPRESSED IN RESPONSE TO VIGNETTES		All	Some Concern	Little/No Concern
**Cancer** vignette				
**Concern about users' Twitter activity being monitored as described above for the purpose of recruitment into a clinical trial**		603 (100%)	300 (49.8%)	283 (46.9%)
**Factors that most impacted level of concern about the above scenario** (respondents could select up to 2)				
	Nature of the disease/medical condition being monitored for	163 (27.0%)	85 (28.3%)	76 (26.9%)
	Whether the text of a Twitter message was monitored vs. a hashtag	152 (25.2%)	92 (30.7%)	54 (19.1%)
	How far back in your Twitter history the researchers might look	167 (27.7%)	89 (29.7%)	74 (26.1%)
	Who is doing the research	284 (47.1%)	136 (45.3%)	140 (49.5%)
	Whether a human being or a computer program is analyzing your Twitter messages	148 (24.5%)	78 (26.0%)	67 (23.7%)
	Use of Twitter as a method in which the researchers contacted you	162 (26.9%)	85 (28.3%)	69 (24.4%)
**Comfort level if a researcher approached them in person at a relevant medical facility**				
	More comfortable in-person	176 (29.2%)	114 (38.0%)	60 (21.2%)
	Neither more or less comfort	209 (34.7%)	85 (28.3%)	115 (40.6%)
	Less comfortable in-person	137 (22.8%)	61 (20.3%)	75 (26.5%)
	Don't know	81 (13.4%)	40 (13.3%)	33 (11.7%)
**Obesity** vignette				

**CONCERN EXPRESSED IN RESPONSE TO VIGNETTES**		**All**	**Some Concern**	**Little/No Concern**
**Concern about users' Twitter activity being monitored as described above for the purpose of recruitment into a clinical trial**		603 (100%)	299 (49.6%)	280 (46.4%)
**Factors that most impacted level of concern about the above scenario** (respondents could select up to 2)				
	Nature of the disease/medical condition being monitored for	169 (28.0%)	88 (29.4%)	77 (27.5%)
	Whether the text of a Twitter message was monitored vs. a hashtag	149 (24.7%)	89 (29.8%)	55 (19.6%)
	How far back in your Twitter history the researchers might look	147 (24.4%)	78 (26.1%)	61 (21.8%)
	Who is doing the research	286 (47.4%)	131 (43.8%)	148 (52.9%)
	Whether a human being or a computer program is analyzing your Twitter messages	123 (20.4%)	66 (22.1%)	52 (18.6%)
	Use of Twitter as a method in which the researchers contacted you	187 (31.0%)	108 (36.1%)	72 (25.7%)
**Comfort level if a researcher approached them in person at a relevant medical facility**				
	More comfortable in-person	161 (26.7%)	92 (30.8%)	65 (23.2%)
	Neither more or less comfort	225 (37.3%)	98 (32.8%)	124 (44.3%)
	Less comfortable in-person	144 (23.9%)	75 (25.1%)	63 (22.5%)
	Don't know	73 (12.1%)	34 (11.4%)	28 (10.0%)
**CONCERN EXPRESSED IN RESPONSE TO VIGNETTES**		**All**	**Some Concern**	**Little/No Concern**
**HPV** vignette				
**Concern about users' Twitter activity being monitored as described above for the purpose of recruitment into a clinical trial**		603 (100%)	298 (49.4%)	276 (45.8%)

**Factors that most impacted level of concern about the above scenario** (respondents could select up to 2)				
	Nature of the disease/medical condition being monitored for	202 (33.5%)	110 (36.9%)	86 (31.2%)
	Whether the text of a Twitter message was monitored vs. a hashtag	162 (26.9%)	95 (31.9%)	62 (22.5%)
	How far back in your Twitter history the researchers might look	126 (20.9%)	62 (20.8%)	59 (21.4%)
	Who is doing the research	271 (44.9%)	132 (44.3%)	130 (47.1%)
	Whether a human being or a computer program is analyzing your Twitter messages	122 (20.2%)	63 (21.1%)	54 (19.6%)
	Use of Twitter as a method in which the researchers contacted you	173 (28.7%)	102 (34.2%)	63 (22.8%)
**Comfort level if a researcher approached them in person at a relevant medical facility**				
	More comfortable in-person	169 (28.0%)	103 (34.6%)	62 (22.5%)
	Neither more or less comfort	233 (38.6%)	87 (29.2%)	141 (51.1%)
	Less comfortable in-person	136 (22.6%)	78 (26.2%)	50 (18.1%)
	Don't know	65 (10.8%)	30 (10.1%)	23 (8.3%)
**HIV/AIDS** vignette				
**CONCERN EXPRESSED IN RESPONSE TO VIGNETTES**		**All**	**Some Concern**	**Little/No Concern**
**Concern about users' Twitter activity being monitored as described above for the purpose of recruitment into a clinical trial**		603 (100%)	349 (57.9%)	228 (37.8%)
**Factors that most impacted level of concern about the above scenario** (respondents could select up to 2)				
	Nature of the disease/medical condition being monitored for	243 (40.3%)	165 (47.3%)	73 (32.0%)

	Whether the text of a Twitter message was monitored vs. a hashtag	154 (25.5%)	96 (27.5%)	51 (22.4%)
	How far back in your Twitter history the researchers might look	133 (22.1%)	87 (24.9%)	38 (16.7%)
	Who is doing the research	250 (41.5%)	131 (37.5)	109 (47.8%)
	Whether a human being or a computer program is analyzing your Twitter messages	127 (21.1%)	81 (23.2%)	45 (19.7%)
	Use of Twitter as a method in which the researchers contacted you	190 (31.5%)	126 (36.1%)	56 (24.6%)
**Comfort level if a researcher approached them in person at a relevant medical facility**				
	More comfortable in-person	174 (28.9%)	122 (35.0%)	50 (21.9%)
	Neither more or less comfort	213 (35.3%)	92 (26.4%)	115 (50.4%)
	Less comfortable in-person	156 (25.9%)	102 (29.2%)	50 (21.9%)
	Don't know	60 (10.0%)	33 (9.5%)	13 (5.7%)
**Smoking** vignette				
**CONCERN EXPRESSED IN RESPONSE TO VIGNETTES**		**All**	**Some Concern**	**Little/No Concern**
**Concern about users' Twitter activity being monitored as described above for the purpose of recruitment into a clinical trial**		603 (100%)	255 (42.3%)	332 (55.1%)
**Factors that most impacted level of concern about the above scenario** (respondents could select up to 2)				
	Nature of the disease/medical condition being monitored for	163 (27.0%)	72 (28.3%)	90 (27.1%)
	Whether the text of a Twitter message was monitored vs. a hashtag	146 (24.2%)	69 (27.2%)	74 (22.3%)
	How far back in your Twitter history the researchers might look	143 (24.7%)	70 (27.6%)	69 (20.8%)

	Who is doing the research	269 (44.6%)	110 (43.3%)	155 (46.7%)
	Whether a human being or a computer program is analyzing your Twitter messages	123 (20.4%)	63 (24.8%)	57 (17.2%)
	Use of Twitter as a method in which the researchers contacted you	173 (28.7%)	89 (35.0%)	78 (23.5%)
**Comfort level if a researcher approached them in person at a relevant medical facility**				
	More comfortable in-person	161 (26.7%)	94 (37.0%)	64 (19.3%)
	Neither more or less comfort	267 (44.3%)	93 (36.6%)	172 (51.8%)
	Less comfortable in-person	122 (20.3%)	47 (18.5%)	72 (21.7%)
	Don't know	52 (8.4%)	20 (7.9%)	24 (7.2%)
