# Supplementary material for: Public Concern About Monitoring Twitter Users and Their Conversations to Recruit for Clinical Trials: Survey Study
Source: J Med Internet Res. 2019 Oct 30;21(10):e15455. doi: 10.2196/15455 (PMC6914244; doi:10.2196/15455)
Supplement: Multimedia Appendix 12 [file jmir_v21i10e15455_app12.pdf]

Multimedia Appendix 12: Select sample of respondents’ comments in response to vignettes.

**COMMENTS**	
**Cancer** vignette: A *cancer research team* at a major *research university* is looking for participants to take part in a clinical trial. They use a software tool that automatically search the past 2 years of Twitter activity to locate accounts that mentioned “*cancer*” anywhere within the text of a Twitter message. Once a relevant message is identified, the research team sends a public “@reply” to the original message, asking if the user is interested in participating in the clinical trial.	
**Less or not concerned**	
	“As a cancer survivor clinical trials would be very helpful for a person just starting their journey.”
	“I think whatever methods are available to help reach people that may need help--should be used.”
	“I am comfortable with whatever.”
	“Not too concerned because being public about this wouldn't be too bad if I'm posting it already.”
	“I think as long as your profile is public, then what you post is visible to anybody and they have access and freedom to respond to you as they see fit.”
	“I have been in a clinical trial for IBS and I am ok with the concept of being a clinical trial participant.”
	“I like doing medical research and do not mind being contacted via Twitter by an individual or group doing research.”
	“I feel like I would be less comfortable with a face to face interaction because it seems more like an invasion of my privacy than online.”
	“If someone would to approach me as I leave a clinic I would be very uncomfortable because it would seem like they are waiting for you. Whereas on Twitter your language is subjecting you to things.”
	“I've never been too fussy over being solicited. I can always say no, I'm not interested, although I know for some people it is very annoying. And, I feel when you post anything publicly on social media, you are basically announcing it to the world, therefore opening yourself up to a lot of attention you probably would not get otherwise.”
	“If you are unconcerned about posting on social media, then you should not be concerned if you are contacted by anyone regarding your posts.”
	“The main reason I am not too concerned is because when I do post to social media it is meant for public. Otherwise I don't post.”
	“If you use the hashtag you are wanting your message to be grouped in to that hashtag grouping. In other words you want it to be seen. I would be down right irate if someone approached me at a doctor's office or hospital as I left an appointment and asked me to participate in a research trial.”

	“If a company had a clinical trial and it was a trial or service that I may need, I would have no problem being contacted.”
	“I always assume everything I post is potentially being monitored.”
**Expressing concern**	
	“I don't think the reply should be public maybe a direct message would be best.”
	“I'd be more comfortable in person because online you don't know who is actually behind the keyboard.”
	“For the purpose of research for cancer I'm ok with this.. but I'd want to know if the data they are collecting on Twitter users is being stored and whether or not it will be shared/sold.”
	“Twitter monitoring is fine, but medical issues are private and people shouldn't be invited with a public tweet.”
	“For something as serious as cancer, I'd prefer to be notified in person about a clinical study. If it were something not major, for example dry skin, then a twitter approach is acceptable.”
	“I dislike the loss of personal privacy in the online arena. I dislike the idea of searching through whatever is posted online for any real purpose.”
	“I do not use social media for the very reasons you are asking these questions.
	“I don't have too much of a problem with the monitoring - it is the method of contact that concerns me. I would not want an employer or potential employer to get the impression that I have a medical issue. ”
	“Social media should never be used to detect medical issues related to a drug trial. Violates HIPPA.”
	“This is why social media is stupid and people should not use it.”
**Obesity** vignette: *Scientists at a pharmaceutical company* are looking for participants to take part in a clinical trial to test a drug designed to treat *obesity*. They use a software tool that allows them to automatically search the past 6 months of Twitter activity to locate accounts that mentioned “obesity”, “overweight” or “lose weight” within the text of a Twitter message. Once a relevant message is identified, the scientists send a public “@reply” to the original message, asking if the user is interested in participating in the clinical trial.	
**Less or not concerned**	
	“I think it’s awesome that medical companies have caught up with times.”
	“This doesn't bother me as much as the cancer monitoring for some reason.”
	“Everyone that uses social media has to know that nothing is really private--but would they believe that the researcher is real?”
	“They should ask doctors to discreetly ask patients that fit into this category if they have

	any interest in losing weight and/or participating.”
**Expressing concern**	
	“Being that I am obese, it’s very hard to be judged in person.”
	“I do not like the idea of my privacy not protected ”
	“I do not want to worry that something I posted is going to be monitored by any entity other than whom it was intended. Also, if companies use these tactics on a regular basis, eventually people will catch on a purposely post things that are not true just to try to get something they may not be entitled to.”
	“I do not want to be approached in person by a random stranger at any time. If you make a public comment then you can't be upset when people see it.”
	“I think it's rather inappropriate to 'bombard' someone with weight loss trials they didn't set out to seek.”
	“I would be highly mad/upset if I was approached publicly for a clinical trial on obesity.”
	“I think the public reply instead of a dm [direct message] could be embarrassing.”
	“I would feel much more at ease about someone replying to a public post I had made about my weight, than for someone to approach me in person and ask me if I'd like to participate in research for obesity. They might as well be calling me 'fatso' right to my face. I would find that embarrassing, but on Twitter, not at all especially after I had put that info out there.”
	“Obesity, while a serious health concern, is also a touchy social subject. Some people may be upset about being 'targeted' this kind of trial.”
**HPV** vignette: A health officer at a state public health office is looking for participants to take part in a study to understand adoption of the HPV vaccination against cervical cancer. The health officer uses a software tool that allows them to search 2 years of Twitter activity to locate accounts that ever included the hashtag “#HPV” or “#HPVvaccine” or “cervical cancer prevention” in a Twitter message. Once identified, the health officer sends a public “@reply” to the original message, asking if the user is interested in participating in the clinical trial.	
**Less or not concerned**	
	“If you make public comments then you cannot get upset by who sees the comment.”
	“For something like HPV , I am okay with them doing either one.”
	“If the person/company reaching out via Twitter is professional, friendly, and provides all the necessary information so they can be checked out, I don't think most people would be offended. If perhaps a website link is included in the message so the target can get all their questions answered in their own time and in private, then I don't think it's a big deal.”
	“If those people are using that hashtag, they obviously care about that cause and should be willing to help.”

	“This type of monitoring maybe welcomed by people who do not know their illness could be taken care of thru a free clinical trial.”
	“Would not be too affected as it is just about the vaccine.”
**Expressing concern**	
	“I feel like my privacy would be invaded, yes I posted it publicly but only to those that follow me, not the whole world, but then again, what is on the internet is public.”
	“How would they know you had vaccine unless violated HIPPA?”
	“The nature of this can be very embarrassing and a public reply could be damaging.”
**HIV/AIDS** vignette: A research team at a major research university is looking for participants to take part in a clinical trial investigating treatment options for *HIV/AIDS*. A team member manually searches Twitter activity to locate accounts that used the hashtags “#HIV”, “#AIDS”, or “#SexwithMen” within the last 3 months. Once identified, the researchers send a public “@reply” to the original message, asking if the user is interested in participating in the clinical trial.	
**Less or not concerned**	
	“As long as the person or researcher making contact with the target is being very transparent about the source of the research and is happy to give information to verify their identity and intent, I wouldn't be alarmed or put off.”
	“I am comfortable with any research.”
	“I have no worries for this but not sure anyone would want to be contacted this way.”
	“I would give almost anything to assist in finding vaccines and treatment for this disease process--it has gone on too long in the world.”
	“If you talk about HIV/Aids on Twitter or any social media, you have to know it's not private.”
**Expressing concern**	
	“This condition definitely need to be addressed privately and not through a public reply.”
	“AIDS and HIV are still hot topics.”
	“On Twitter users are using the specific language. These users have already disclosed their opinions or diagnosis. I feel like it's similar to outing someone on accident if a company were to just randomly ask people.”
	“HIV a very serious and private disease... it is something that needs to be discussed in person.”
	“This is completely inappropriate for this topic.”

**Smoking** vignette: A health officer at a local public health office is looking for participants to take part in a study to understand the smoking habits of local citizens. The health officer uses a software tool that allows them to search the past 2 years of Twitter activity to locate accounts that mentioned words such as “smoking”, “cigarette”, “e-cigarette” or “vaping" within the text of a Twitter message. Once a relevant Twitter message is identified, the health officer sends a public “@reply” to the original message, asking if the user is interested in participating in the clinical trial.	
**Less or not concerned**	
	“Being a former smoker, research is necessary.”
	“Smoking is a lifestyle choice, so it isn't as personal as a disease.”
	“People need help to battle tobacco addiction. I've only been off cigarettes for 6 months...would have appreciated being approached with help to quit.”
	“This one would be OK with the public response.”
	“In this case, the issue at hand for the study is more common among others and I don't see it to be as personal.”
	“The topic is not that sensitive to me so the question would not bother me.”
	“I feel as though smoking or vaping is a very popular act so it would be comfortable in either situation.”
	“This is an open topic. there really wouldn't be too much with exposing anyone here.”
	“This is not an embarrassing issue and I don't see a problem utilizing this method.“
**Expressing concern**	
	“The in person approach is very intimidating and can be somewhat insulting. If I were approached in person, I would feel as though the researcher has been not only watching me, but also making preconceived judgements about me.”
	“I'm really not comfortable with sending public reply messages. I think it's a big reach.”
	“It's an invasion of privacy to monitor this and should be voluntary.”
	“I would like to take part in this but not be contacted by Twitter.”
